# In-sewer microplastics drive microbial metabolic shifts toward enhanced methanogenesis

**DOI:** 10.1016/j.ese.2026.100726

**Published:** 2026-06-20

**Authors:** Yaxin Wang, Xiuhong Liu, Zhipeng Zhang, Ruxian Jing, Xiaoyin Zhao, Weipeng Han, Chenduo Huang, Qing Yang

**Affiliations:** a Key Laboratory of Beijing for Water Quality Science and Water Environment Recovery Engineering, Beijing University of Technology, Beijing, 100124, China; b National Engineering Laboratory for Advanced Municipal Wastewater Treatment and Reuse Technology, Beijing University of Technology, Beijing, 100124, China; c Beijing Waterworks Group Co., Ltd, Beijing, 100031, China

**Keywords:** Microplastics, Sewer systems, Hydroxyl radicals, Aging mechanism, Oxidative stress, Functional metabolism

## Abstract

Microplastics (MPs) in sewer systems can be transported extensively before entering wastewater treatment plants. Sewer systems harbor complex microbial communities under low-oxygen, sulfide-rich conditions that drive key biogeochemical cycles. These conditions drive microplastic aging, whereas these particles concurrently perturb sewer microbial ecology and metabolic functions. However, the underlying mechanisms of in-sewer microplastic aging and their subsequent impacts on sewer microbiomes remain unclear. Here we show that hydroxyl radicals preferentially attack ester bonds (C–O) in polyethylene terephthalate (PET) and polybutylene adipate terephthalate (PBAT) MPs, increasing surface roughness, reducing particle size, promoting surface oxidation, and ultimately inducing polymer chain scission. Exposure to PET and PBAT MPs at 30–500 particles L^−1^ intensified oxidative stress, disrupted membrane integrity and permeability, impaired microbial activity, and suppressed sulfide production in a dose-dependent manner. These disruptions coincided with weakened microbial co-occurrence networks and a shift from stochastic toward deterministic community assembly. High doses of PET and PBAT MPs reduced hydrolytic/fermentative bacteria and sulfate-reducing bacteria by up to 63.4% and 49.7%, respectively, while enriching hydrogen-producing acetogenic bacteria and methanogenic archaea by 48.4–67.0%, consistent with reduced sulfidogenic potential and enhanced methanogenic potential. Changes in genes related to antioxidant defense, SOS response, quorum sensing (e.g., *sod*A, *kat*G, *lex*A, and *lux*S), and redox signaling suggested potential mechanisms of microbial metabolic perturbations aggravated by PET and PBAT MPs. Our results indicate that sewer systems are not passive conduits but active reactors that promote MP aging, and that MPs reshape microbial functions. Microplastic control may therefore help reduce downstream particle pollution and limit perturbations to urban sewage biogeochemistry.

## Highlights

• Urban sewers act as active bioreactors that accelerate microplastic aging.
• Hydroxyl radicals drive polymer chain scission by attacking ester bonds.
• High microplastic loading reduces sulfate-reducing bacteria by up to 49.7%.
• Microplastic exposure enhances methanogenesis while suppressing sulfidogenesis.

## Graphical abstract

**Figure:**
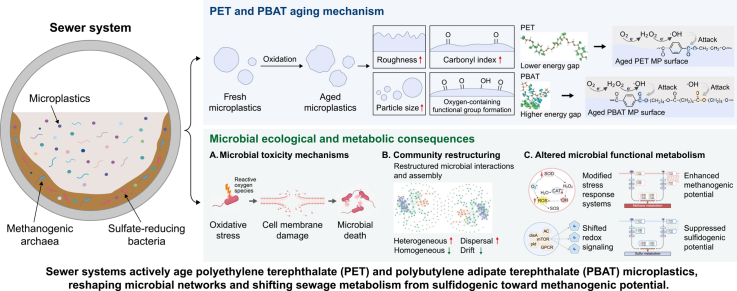

## 1 Introduction

Microplastics (MPs) are persistent pollutants of global concern that have the potential to threaten ecosystems and human health [1]. A substantial volume of MPs (10–470 particles L^−1^) [2] originating from domestic sewage, industrial wastewater, and stormwater runoff is transported via urban sewer systems, which are extensive, complex underground networks where diverse physical, chemical, and biological processes occur. Such particles inevitably interact with microbiomes in sewers characterized by low dissolved oxygen (DO), abundant nutrients, and active microbial populations. Their physicochemical properties are therefore altered by the complex environment in sewer systems through biofilm colonization and biochemical reactions. Despite these realities, research on MPs has predominantly focused on their transport, the efficiency of their removal, and their ecotoxicological effects in wastewater treatment plants (WWTPs) [[3], [4], [5]]. Studies on interactions between MPs and sewer ecosystems have also been lacking, even though these systems constitute a significant proportion of urban drainage networks.

Research on the aforementioned issues is important because sewers are not merely passive conduits but dynamic tubular bioreactors that harbor various microbial communities and mediate complex biogeochemical processes [6]. Although direct evidence regarding the effects of MPs on sewer microbiomes remains limited, studies in functionally related anaerobic or redox-active microbial systems, including anaerobic digestion systems, anaerobic granular sludge, and coastal sediments, have shown that MPs can disrupt microbial community structure and impair core metabolic pathways involved in biogeochemical transformations [[7], [8], [9]]. These findings suggest that sewer microbial communities, which develop under low-oxygen, nutrient-rich, and microbially active conditions, may also be vulnerable to MP exposure. This inference is further supported by recent studies showing that sewer microbial communities are sensitive to exogenous contaminants (e.g., tetracycline and minocycline), which reduce microbial abundance and impair key metabolic pathways involved in the production of methane and hydrogen sulfide [10,11]. On this basis, MPs, as another class of contaminants transported through sewer systems, may also pose ecological risks to sewer microbiomes. Unlike antibiotics, which act primarily as soluble toxicants that inhibit enzyme activities or compromise genetic stability, MP-induced stress involves more complex physicochemical pathways [12]. MPs and their surface biofilms can reshape the local microenvironment by modifying biofilm structure and mass-transfer dynamics [13]. Furthermore, these particles and the byproducts resulting from their aging can compromise membrane integrity and increase the production of intracellular reactive oxygen species (ROS), thereby potentially disrupting microbial ecological functions [14].

The nonrandom co-occurrence patterns and interactions among taxa are commonly identified through network analysis [15]. In parallel, community assembly analysis can help distinguish between the deterministic processes (e.g., environmental selection) and stochastic processes that shape community structures [16]. Together, these approaches can clarify how MP exposure reshapes the structures and functioning of microbial communities through altered interactions and assembly mechanisms. The problem is that the ecological impacts of MPs on microbial community structures, interspecies interactions, assembly processes, and the metabolic potential of sewer microbiomes have been inadequately elucidated.

Sewer-specific conditions, including long transport distances, fluctuating redox gradients, and abundant reactive constituents (e.g., sulfides, iron, and natural organic matter), may further influence the physicochemical transformation of MPs. Studies have indicated that sulfides can generate ROS (e.g., hydroxyl and superoxide radicals) via auto-oxidation, which can subsequently accelerate MP aging [17]. In turn, the redox reactions occurring on aged MP surfaces might promote ROS generation, creating a self-reinforcing aging cycle. The environmental aging of biodegradable MPs has also elicited increasing attention. Such particles can age through microbial action, enzymatic hydrolysis, or redox reactions, often faster than traditional MPs [18,19]. This process alters the physicochemical properties of such particles and may release intermediate products that affect surrounding ecosystems. Nevertheless, no study has accounted for the mechanisms by which sewer-derived MPs—especially biodegradable MPs—age and the impact of hydroxyl radicals (·OH) generated during MP aging on sewer microbial metabolism.

To address the aforementioned deficiencies, we constructed laboratory sewer reactors using concrete sewer sections (over 10 years old) collected from municipal networks and fed with raw domestic sewage. Two typical materials used in MP studies, polyethylene terephthalate (PET) and biodegradable polybutylene adipate terephthalate (PBAT), were systematically investigated regarding their bidirectional interactions with sewer ecosystems. The objectives were to (1) examine the drivers and potential mechanisms governing the aging of PET and PBAT MPs; (2) determine how MPs reshape microbial interactions and assembly mechanisms using co-occurrence network analysis and null model-based community assembly analysis and link these ecological shifts to changes in metabolic potential; and (3) identify the mechanisms by which MPs plausibly affect microbial community and metabolic functions. In pursuing these objectives, this study offers valuable insights into the fate of MPs in sewers, informs wastewater management and operational strategies for controlling MP loads, and provides scientific evidence for developing MP source-control regulations and environmental standards for urban drainage systems.

## 2 Materials and methods

### 2.1 Preparation of MPs

Virgin PET and PBAT MPs were purchased from Tesulang Chemical Materials Co., Ltd. (Dongguan, China) to analyze their morphological characteristics and particle-size distribution using scanning electron microscopy (SEM; SU8600, Hitachi, Japan) (Supplementary Fig. S1a). The SEM images showed that the MPs were mainly granular, with average particle sizes of 210 ± 33 μm for PET and 238 ± 30 μm for PBAT. These sizes fall within the size range typically observed for MPs in WWTP influent [20]. Representative particle sizes were selected to reduce size-related variability and better isolate concentration-dependent effects. The chemical compositions of the MPs were then confirmed through attenuated total reflectance Fourier transform infrared (FTIR) spectroscopy (IS50, Thermo Fisher Scientific, USA) (Supplementary Fig. S1b).

### 2.2 Setup and operation of sewer reactors

A reinforced concrete sewer pipe (400 mm inner diameter), previously used in a municipal sewer system, was sectioned into two 300 mm segments to construct laboratory-scale sewer reactors, and seed sludge was collected from upstream inspection wells in urban sewer systems. The reactors, with a gas–liquid–solid ratio of 2:2:1 and an effective liquid volume of 20 L, were used to simulate the operation of gravity-flow sewer systems. Domestic sewage was used as the influent, with a hydraulic retention time of 8 h (Supplementary Table S1), within the range observed in field sewer systems [21]. A top-mounted stirrer was operated at 40 rpm to replicate the hydraulic shear stress typical of gravity-flow sewers [22].

We conducted a 120-day long-term MP exposure experiment comprising sequential phases. In phase I (days 0–30), all reactors were fed with raw domestic sewage. During phases II–IV (days 31–60, 61–90, and 91–120, respectively), domestic sewage containing PET or PBAT MPs at 30, 100, and 500 particles L^−1^ was introduced into the sewer reactors. These concentrations were selected to represent environmentally relevant low, intermediate, and elevated loading scenarios, as MP abundance in municipal WWTP influent can vary substantially over time among systems [2,[23], [24], [25]]. Mass-based MP concentrations were estimated from particle number concentrations by assuming spherical particles [26]: $C_{m}=C_{n}\times \frac{\pi d^{3}}{6}\times \delta_{p}$, where $C_{m}$ is the mass concentration; $C_{n}$ is the particle number concentration; *d* is the mean particle diameter; and $\delta_{p}$ is the polymer density, 1.4 g cm^−3^ for PET and 1.3 g cm^−3^ for PBAT. Operational parameters were kept consistent across all the phases.

### 2.3 Setup of MP aging experiments

This study also involved two separate aging experiments. To simulate the aging characteristics of MPs in sewer systems, the PET and PBAT MPs were placed in high-permeability nylon mesh bags (125 μm) and introduced into the reactors. The mesh size effectively prevented MP loss while allowing sewage, nutrients, and microorganisms to freely penetrate the material, thereby facilitating continuous interactions with MP surfaces. MPs were collected on days 15 and 30 for physicochemical characterization. Simultaneously, the chemical mechanism of MP aging in sewers was investigated through separate batch experiments conducted in serum bottles. Specifically, 12.5 mg of PET and PBAT MPs were added to 250 mL of sewage or deoxygenated Milli-Q water with a DO concentration of approximately 0.02–0.09 mg L^−1^, as measured using WTW DO meters (3460, WTW, Germany). The serum bottles were then sealed, placed on a shaker set to 160 rpm at 25 °C, and run for 30 days in the dark. To clarify the role of ROS in MP aging, 500 mM isopropanol (IPA) [[27], [1]] mM p-benzoquinone (PBQ) [28] were added to each of the serum bottles as specific scavengers of ·OH and superoxide radical anions (O_2_^·−^) for mechanism verification. Finally, the PET and PBAT MPs were washed with ethanol and Milli-Q water and dried at 40 °C for analysis.

### 2.4 Other procedures

We employed standard methods [29] to determine the concentrations of soluble chemical oxygen demand (SCOD), phosphate (PO_4_^3−^-P), ammonia nitrogen (NH_4_^+^-N), and sulfide in the sewage. Extracellular polymeric substances (EPSs) were extracted, and their components were determined as described in previous studies [30,31] (Supplementary Text S1). Intracellular ROS levels, lactate dehydrogenase (LDH) release, and superoxide dismutase (SOD) and catalase (CAT) activity were quantified using enzyme-linked immunosorbent assay kits (Supplementary Text S2). Flow cytometry with dual-fluorescent staining was performed to assess bacterial viability and metabolic activity (Supplementary Text S3) [32], while atomic force microscopy (AFM; Dimension FastScan, Bruker, Germany) and SEM were conducted to examine MP morphology. AFM was also carried out to measure surface roughness. The MP particles in three SEM images were counted using ImageJ, with a minimum of 100 particles per image, to determine the particle size distribution. Two-dimensional correlation spectroscopy (2D-COS) was performed on FTIR spectra, with aging time as the external perturbation, to track shifts in MP functional groups during aging [33]. The carbonyl index, defined as the ratio of the carbonyl band absorbance to that of the reference band (methyl groups), was regarded as an indicator of MP aging [34]. Electron paramagnetic resonance (EPR) spectroscopy (A300-10/12, Bruker, Germany) was conducted to quantify the ROS generated in the MP suspensions during aging. The spin-trapping agent for ·OH and O_2_^·−^ in water or dimethyl sulfoxide was 5,5-dimethyl-1-pyrroline-N-oxide (DMPO). The detailed operating procedures and EPR settings are provided in Supplementary Text S4.

### 2.5 16S rRNA amplicon and metagenomic sequencing

Sludge samples were collected from the reactors and lyophilized in a freeze dryer (FreeZone, Labconco, USA) for 72 h. Approximately 50–100 mg of the lyophilized sludge was weighed, after which DNA was extracted from it using a FastDNA Spin Kit for Soil (MP Biomedicals, USA) according to the manufacturer's instructions. Illumina MiSeq and NovaSeq sequencing were conducted by Majorbio Bio-Pharm Technology Co., Ltd. (Shanghai, China). Bacterial and archaeal 16S rRNA genes were amplified using primers 515FmodF and 806RmodR, while DADA2 was used to cluster clean reads into amplicon sequence variants (ASVs). ASV taxonomy was assigned using a naïve Bayes classifier trained on the SILVA database (70% confidence threshold). The sequencing data from multiple mixed assemblies were processed with MEGAHIT, and METAGENE was used to detect open reading frames in assembled sequences with overlaps longer than 100 bp. A nonredundant gene catalog was constructed by clustering predicted genes using CD-HIT (90% coverage threshold). Following alignment to the nonredundant gene catalog through SOAPaligner (95% identity threshold), high-quality reads were then functionally profiled. Functional annotation was conducted using DIAMOND for homology searches against the Kyoto Encyclopedia of Genes and Genomes (KEGG). Functional genes were identified based on KEGG Orthology identifiers, with the annotation results used as the reference.

For 16S rRNA gene amplicon sequencing, which included community assembly and co-occurrence network analyses, each treatment involved three to six independent biological replicates. Metagenomic sequencing was conducted using two independent biological replicates per treatment. To mitigate local heterogeneity and improve the representativeness of the microbial communities forming in the sewage system that we built, each biological replicate was a composite sample prepared by pooling and homogenizing three to five subsamples collected from distinct locations in the reactors.

### 2.6 Calculations based on density functional theory

To identify surface sites on the PET and PBAT MPs susceptible to radical attack, we calculated Fukui functions using Multiwfn. The Fukui functions for nucleophilic (*f*^+^), electrophilic (*f*^−^), and radical (*f*^0^) attacks were calculated as follows:

(1) $$f^{+}\left(x\right)=\rho_{N+1}\left(x\right)-\rho_{N}\left(x\right)$$

(2) $$f^{-}\left(x\right)=\rho_{N}\left(x\right)-\rho_{N-1}\left(x\right)$$

(3) $$f^{0}\left(x\right)=\frac{f^{+}\left(x\right)+f^{-}\left(x\right)}{2}=\frac{\rho_{N+1}\left(x\right)-\rho_{N-1}\left(x\right)}{2}$$

where *N* denotes the number of electrons in the neutral system, ***x*** denotes the position vector in three-dimensional real space at which the electron density and local Fukui function are evaluated; and $\rho_{N}\left(x\right)$, $\rho_{N+1}\left(x\right)$, $\rho_{N-1}\left(x\right)$ are the electron densities of the neutral, anionic, and cationic states, respectively. For each polymer model, these electron densities were calculated at the optimized geometry of the corresponding neutral system.

### 2.7 Data analysis

Co-occurrence networks were constructed from the top 50 most abundant genera across all samples, which together accounted for approximately 70% of the total relative abundance. This filtering step was applied to reduce the influence of rare taxa while retaining the dominant communities in the constructed sewer system. We calculated pairwise Spearman correlations and retained correlations with |*r*| > 0.8 and *p* < 0.05 for network construction. This threshold was chosen to enable focus on relatively strong associations while avoiding an overly sparse network under more stringent conditions.

Network topological properties were calculated using Gephi (v0.10.1). To assess the robustness of the observed network patterns, sensitivity analyses were conducted using the top 100 genera (which account for approximately 79% of the total relative abundance) and a more stringent correlation threshold (|*r*| > 0.9). Although some topological properties varied across the parameter settings, the overall trends in the topological parameters remained consistent (Supplementary Table S2). A null model analysis was carried out to quantify the relative contributions of ecological processes to community assembly in the sewer communities [35], following the framework described in Supplementary Text S5. Statistical analyses were conducted using IBM SPSS (v26.0). Group differences were tested via one-way ANOVA (*p* < 0.05), and results were expressed as mean ± standard deviation.

## 3 Results and discussion

### 3.1 Hydroxyl radicals in the anaerobic sewers drove the aging of PET and PBAT MPs

#### 3.1.1 Physical and chemical aging

**Fig. 1:**
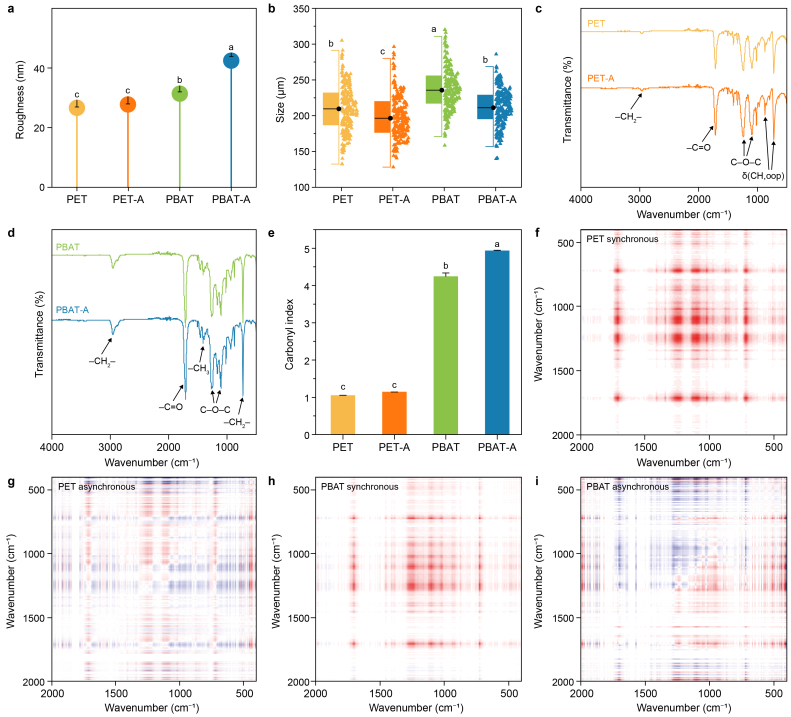
Changes in MP characteristics during aging in sewers. **a**–**b**, Surface roughness (**a**), particle size distribution (**b**) of PET and PBAT MPs. Black circles denote mean values, boxes denote the interquartile range (25–75%), whiskers denote the data range, and points denote individual measurements and potential outliers. **c**–**d**, Fourier transform infrared spectra of pristine and aged PET (**c**) and PBAT (**d**) MPs. **e**, Carbonyl indices of pristine and aged PET and PBAT MPs. Error bars denote the standard deviation. Groups sharing at least one common letter do not differ significantly, whereas groups with no letters in common differ significantly (*p* < 0.05). **f**–**i**, Synchronous (**f**, **h**) and asynchronous (**g**, **i**) two-dimensional correlation spectroscopy of aged PET (**f**, **g**) and PBAT (**h**, **i**) MPs. PET: polyethylene terephthalate; PBAT: polybutylene adipate terephthalate; MPs: microplastics. PET-A and PBAT-A denote aged PET MPs and aged PBAT MPs, respectively.

The physical (e.g., morphology, size distribution) and chemical (e.g., functional groups) properties of the PET and PBAT MPs changed after *in situ* aging in the reactors. More specifically, their surface roughness increased by 1.5% and 26.6%, respectively (Fig. 1a). The AFM images showed distinct surface features on the aged PBAT MPs (e.g., folds and furrows), whereas only minor morphological changes were observed on the PET counterparts (Supplementary Fig. S2). The particle diameter of the PET MPs slightly decreased, whereas that of the PBAT MPs declined by 11.3% (Fig. 1b), indicating the preferential fragmentation of the biodegradable PBAT MPs.

The FTIR spectroscopy confirmed the chemical modification of PET and PBAT MPs (Fig. 1c). The aged PET MPs exhibited intensified bands at 1710 cm^−1^ (*v*C=O stretching) and 1240/1090 cm^−1^ (*v*C–O–C stretching vibration), suggesting the formation of oxygen-containing functional groups after aging in the reactors. Similar enhancements were observed for the PBAT MPs at 1710 and 1260/1100 cm^−1^ (*v*C–O–C stretching vibration), but they were accompanied by a greater increase in carbonyl indices (16.3%) than that found in the PET MPs (9.2%) (Fig. 1e). These results are consistent with the higher environmental reactivity of PBAT MPs, likely attributable to their lower crystallinity and more labile aliphatic ester domains compared with the aromatic backbone of PET MPs.

The 2D-COS analysis clarified the intrinsic causes of MP aging in the reactors. On the basis of Noda's rules and the synchronous and asynchronous spectra [36], different degradation patterns were identified in the PET and PBAT MPs (Supplementary Table S3–S4). For the PET MPs, functional group changes occurred in the order C–O (1240 cm^−1^), C=O (1710 cm^−1^), CH_2_ (720 cm^−1^), C–O (1020 cm^−1^), C–O (1100 cm^−1^), and CH_2_ (1410 cm^−1^) (Fig. 1f and g). These changes suggest slow, stepwise oxidative degradation, beginning with cleavage of ester C–O bonds, followed by carbonyl formation and gradual disruption of the polymer backbone (CH_2_). The PBAT MPs exhibited a different degradation sequence: C–O (1020 cm^−1^), C–O (1100 cm^−1^), C–O (1260 cm^−1^), CH_2_ (720 cm^−1^), and C=O (1710 cm^−1^) (Fig. 1h and i). The near-simultaneous early changes across multiple C–O bands suggest rapid and extensive oxidative cleavage in the PBAT MPs, particularly in aliphatic ester segments. These modifications were followed by the oxidation of methylene units and the accumulation of carbonyl-containing groups. The broader spectral range and earlier spectral shifts in the PBAT MPs reflected greater reactivity toward oxidative species, consistent with the observed increases in surface roughness and carbonyl indices as well as the reduction in particle size occurring after sewer exposure. Overall, the sewer conditions accelerated the aging of the PET and PBAT MPs via synergistic physicochemical pathways, particularly for the latter.

#### 3.1.2 Mechanism of hydroxyl radical-mediated aging

**Fig. 2:**
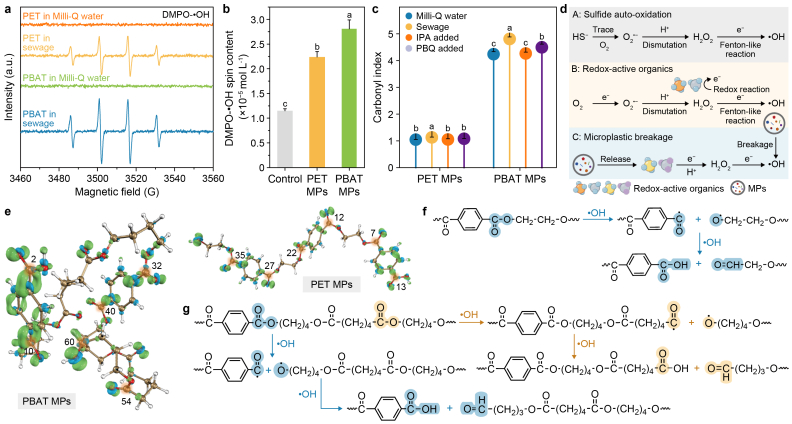
Reactive oxygen species generation and proposed transformation pathways of PET and PBAT MPs in sewers. **a**, Electron paramagnetic resonance spectra of hydroxyl radicals (·OH) for PET and PBAT MPs in Milli-Q water and sewage. **b**, 5,5-Dimethyl-1-pyrroline-N-oxide (DMPO)-·OH spin concentration in sewage alone and sewage containing PET or PBAT MPs. **c**, Carbonyl index of MPs in Milli-Q water, sewage-aged MPs, and sewage-aged MPs in the presence of the ·OH scavenger isopropanol (IPA) or the superoxide radical anion (O_2_^·−^) scavenger p-benzoquinone (PBQ). Error bars denote the standard deviation. Bars labeled with at least one common letter do not differ significantly, whereas bars with no letters in common differ significantly (*p* < 0.05). **d**, Schematic illustration of formation mechanisms of ·OH and O_2_^·−^ in sewers. **e**, Reactive oxygen species attack sites on PET and PBAT MPs calculated by Fukui functions (*f*^0^). Green and blue indicate positive and negative phases of the wave function, respectively. Numbers denote the atom indices of potential ·OH-attack sites. **f**–**g**, Proposed transformation pathways of PET (**f**) and PBAT (**g**) MPs in sewers. PET: polyethylene terephthalate; PBAT: polybutylene adipate terephthalate; MPs: microplastics.

Domestic sewage is rich in natural organic matter, sulfide, and other redox-active compounds. Although DO levels in sewer systems are generally low, trace amounts of O_2_ can still act as electron acceptors, sustaining sulfide autoxidation and subsequent redox reactions that initiate free-radical chain reactions and generate ROS in low-DO environments [17,37]. Therefore, ROS generation is expected in sulfide-rich sewer systems. Consistent with this mechanism, the EPR measurements conducted in this work indicated typical signals for ·OH and O_2_^·−^ in the sewage, but no radical signals were observed in the deoxygenated Milli-Q water (Fig. 2a and Supplementary Fig. S3). Further semi-quantitative EPR analysis revealed that DMPO-·OH spin concentrations were higher in the sewage containing the PET and PBAT MPs than in that without such particles (controls, Fig. 2b). This finding implies that MPs, their surface redox reactions, or leachates enhance the generation of ·OH in sewers. To elucidate the specific roles of ·OH and O_2_^·−^ in MP aging, ·OH and O_2_^·−^ were selectively quenched using IPA and PBQ, respectively. After O_2_^·−^ quenching, the carbonyl indices of the PET and PBAT MPs were slightly elevated over the levels found in pristine MPs, suggesting that although O_2_^·−^ contributes to MP aging, it does not dominate this process (Fig. 2c). Notably, when ·OH was quenched with IPA, the carbonyl indices of the PET and PBAT MPs decreased significantly by 7.9% to 13.5% compared with those observed in sewage-aged MPs, returning to levels similar to those of pristine MPs. These findings demonstrate that ·OH is the primary active species driving the aging of PET and PBAT MPs in sewer systems.

Several processes contributed to ROS generation in the sewer system built in this work (Fig. 2d). First, the auto-oxidation and redox cycling of sulfides could generate ·OH and other oxidants, even in low-DO environments. Second, redox-active organic components could mediate electron transfer to O_2_ and promote the generation of ROS [38]. Third, the fragmentation of the PET and PBAT MPs could directly lead to electron transfer and ·OH production [39]. In addition, the organic matter leaching from the PET and PBAT MPs could generate free radicals and mediate electron transfer to O_2_^·−^, ultimately forming H_2_O_2_ and ·OH through sequential reactions [40]. Although microbial enzymatic degradation and hydrolysis may also have contributed to these effects, these processes were generally slower and required longer timescales to produce significant oxidative signatures on the MP surfaces [41]. Therefore, the carbonyl formation and ester bond cleavage observed in this study are consistent with ROS-driven oxidative processes.

The mechanisms by which the PBAT and PET MPs were degraded were explored through calculations of frontier molecular orbitals and Fukui functions. The PET MPs exhibited a relatively large energy gap between the highest occupied molecular orbital and the lowest unoccupied molecular orbital (Δ*E* = 5.09 eV) (Supplementary Fig. S4). This result indicates strong molecular stability and limited capacity for electron transfer, thereby reducing susceptibility to ROS-induced degradation. Although the *f*^0^ values of carbon atoms on the benzene ring in the PET MPs were comparatively elevated, the intrinsic stability of the aromatic ring and the existence of conjugated π bonds conferred resistance to radical-induced degradation [42]. However, the carbon atoms adjacent to the aromatic ring (e.g., C27, C12, C13, C35, C7, and C22) were more prone to attack by ·OH (Fig. 2e, Supplementary Fig. S5a, and Supplementary Table S5). Thus, the ·OH generated in the sewer environment primarily targeted ester bonds (C–O), thereby initiating radical formation in the PET chain, followed by the formation of oxygen-containing groups. In contrast, the lower energy gap observed in the PBAT MPs (Δ*E* = 4.39 eV) facilitated electron transfer and rendered the polymer more vulnerable to oxidative degradation than the PET MPs. In addition to the C=C on the aromatic ring, several carbon atoms connected to the benzene ring (C10, C40, C60, C2, and C32) and aliphatic chain carbon (C54) exhibited considerable reactivity toward ROS (Fig. 2e, Supplementary Fig. S5b, and Supplementary Table S6). Due to the absence of π-conjugation, the ester bonds in the aliphatic backbone served as primary degradation targets. Taken together, these results show that both PET and PBAT MPs initiate ROS-driven degradation via the cleavage of the ester C–O bond (Fig. 2f and g), resulting in radical intermediates and the subsequent formation of oligomers with hydroxyl and carboxyl groups.

The PBAT MPs exhibited notably faster degradation rates than the PET MPs, primarily due to differences in vulnerable sites and energy band gaps between the two plastics. In summary, the ·OH generated in the sewer system was a major driver of the aging of PET and PBAT MPs (e.g., crack formation, surface roughening, and even breakage). Simultaneously, the aging of the PET and PBAT MPs likely contributed to ROS accumulation in the reactors, thereby exacerbating oxidative stress and potentially affecting the microbial communities.

### 3.2 PET and PBAT MP exposure altered microbial ecological processes

#### 3.2.1 PET and PBAT MPs exacerbated microbial oxidative stress

**Fig. 3:**
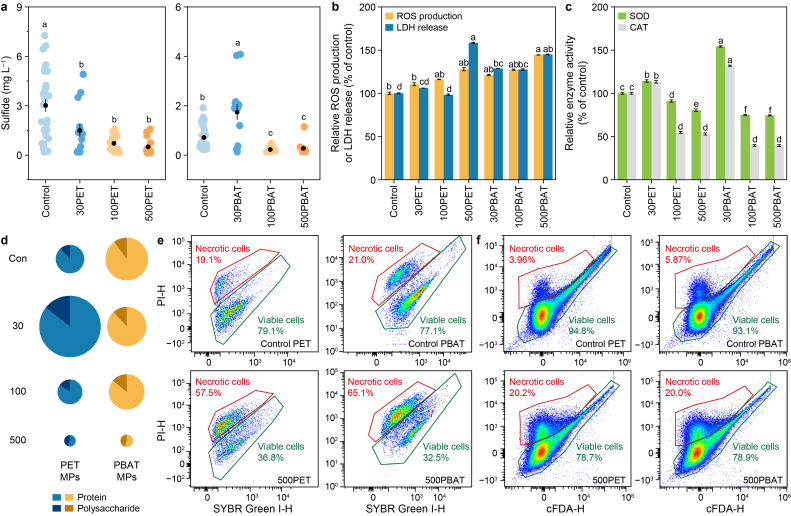
Pollutant transformation and oxidative stress in sewers exposed to PET and PBAT MPs. **a**, Sulfide content after exposure to PET and PBAT MPs. Black circles denote mean values, and black bars denote the quartile (25–75%) lines. **b**, Reactive oxygen species (ROS) production and lactate dehydrogenase (LDH) release. **c**, Activities of superoxide dismutase (SOD) and catalase (CAT). Error bars denote the standard deviation. Bars labeled with at least one common letter do not differ significantly, whereas bars with no letters in common differ significantly (*p* < 0.05). **d**, Variations in protein and polysaccharide in extracellular polymeric substances (EPSs). The size of each pie chart is proportional to the EPS concentration. Con, 30, 100, and 500 denote PET and PBAT MP concentrations of 0, 30, 100, and 500 particles L^−1^, respectively. **e**–**f**, Physiological status of sewer microbial cells stained with SYBR Green I/propidium iodide (PI) (**e**) and 5(6)-carboxyfluorescein diacetate (cFDA)/PI (**f**). PET: polyethylene terephthalate; PBAT: polybutylene adipate terephthalate; MPs: microplastics. The numbers preceding PET and PBAT MPs denote the added particle concentrations.

Relative to the controls, long-term exposure to the PET and PBAT MPs exerted negligible effects on the transformation of SCOD, NH_4_^+^-N, and PO_4_^3−^-P in the sewer system (Supplementary Fig. S6–S7). However, following exposure to PET and PBAT MPs at 500 particles L^−1^, the average sulfide concentrations decreased by 89.5% and 71.9%, respectively, compared with the levels found in the control condition (Fig. 3a). The ROS levels increased with prolonged exposure to PET and PBAT MPs at 30–500 particles L^−1^, indicating that the particles exacerbated oxidative damage in cells (Fig. 3b). Upon sensing elevated levels of oxidative stress, the microbial cells activated enzymatic antioxidant defenses to restore redox homeostasis [43]. Exposure to PET and PBAT MPs at 30 particles L^−1^ triggered the protective function of microbial antioxidant systems, amplifying SOD and CAT activity (Fig. 3c). Such activity, nonetheless, diminished significantly by 8.9% to 25.5% at 100 and 500 particles L^−1^, revealing that microbial antioxidant defense systems are insufficient to resist excessive ROS generation.

The EPSs, the primary defensive barrier against external stressors [10], exhibited a concentration-dependent response to the PET and PBAT MPs. The low-dose of PET and PBAT MPs bound to the EPSs via hydrophobic and π–π interactions, promoting EPS secretion and forming an “eco/bio-corona” that buffered toxicity in the short term [44]. Thus, exposure to PET and PBAT MPs at 30 particles L^−1^ increased EPS production by 48.6% and 17.9%, respectively (Fig. 3d), relative to the controls. In contrast, exposure to PET and PBAT MPs at 100 and 500 particles L^−1^ reduced EPS concentration by 14.9–66.7%, suggesting that excessive MP exposure impairs microbial EPS synthesis and weakens their defensive function. Furthermore, increased LDH release indicated a loss of integrity in the microbial cell membrane (Fig. 3b). This phenomenon was also evidenced by the increased percentage of necrotic cells (38.4% and 44.1%) after exposure to PET and PBAT MPs at 500 particles L^−1^ (Fig. 3e). Since cell membrane integrity only partially represents cellular activity, intracellular metabolic function was assessed by measuring esterase activity via 5(6)-carboxyfluorescein diacetate/propidium iodide staining (Fig. 3f) [45]. The proportion of viable cells with enzymatic activity decreased as the MP dose increased, indicating that PET and PBAT MPs impair intracellular metabolic function. Notably, the proportion of active cells exhibiting esterase activity (78.7% and 78.9%) was significantly higher than that of viable cells (36.9% and 32.5%) exposed to PET and PBAT MPs, respectively. This result reflects that residual metabolic function persists despite cell membrane damage in certain microorganisms.

Consequently, the inhibitory effects of the PET and PBAT MPs on the sewer microorganisms were more likely driven by the synergistic interplay of multiple mechanisms. The aging of PET and PBAT MPs may enhance interfacial contact between MPs and microbes, promote the adsorption and localized enrichment of EPSs and cellular components, and increase the accumulation of reactive species at the MP–microbe interface [44]. Meanwhile, the chemical constituents released during the aging of PET and PBAT MPs may act as dissolved chemical stressors, thereby damaging microbial membrane function and metabolic activity [46]. Additionally, PET and PBAT MPs may enhance the generation and accumulation of ROS, particularly ·OH, thereby exacerbating oxidative stress and triggering a cascade of secondary damage, including disruption of antioxidant systems, membrane destruction, and increased membrane permeability [32]. The loss of membrane integrity can lead to leakage of cytoplasmic components, including esterases, SOD, and CAT, which are subsequently oxidized by reactive species, thereby inactivating metabolism and inducing cell death. In this research, increased membrane permeability likely facilitated the intracellular entry of reactive substances, exacerbating ROS accumulation beyond cellular repair capacity, which in turn promoted membrane dysfunction and microbial necrosis. Therefore, the LDH leakage, esterase inactivation, cellular necrosis, and inhibition of sulfide production observed in this study were potentially the combined result of MP–microbe interactions, released chemical constituents, and oxidative stress.

#### 3.2.2 Shifts in microbial interactions and assembly mechanisms

**Fig. 4:**
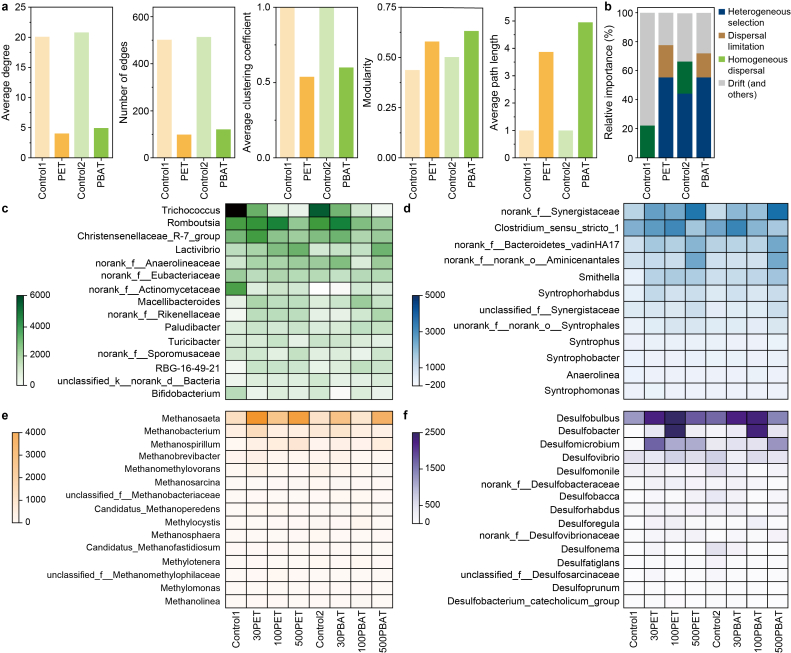
Network topology, community assembly, and shifts in key functional guilds after exposure to PET and PBAT MPs. **a**, Topological parameters of co-occurrence networks. **b**, Relative importance of divergent ecological processes in microbial assembly. **c**–**f**, Relative abundance of hydrolytic/fermentative bacteria (**c**), hydrogen-producing acetogens (**d**), methanogenic archaea (**e**), and sulfate-reducing bacteria (**f**) at the genus level. Control1: the control corresponding to the PET MP exposure; Control2: the control corresponding to the PBAT MP exposure; PET: polyethylene terephthalate; PBAT: polybutylene adipate terephthalate; MPs: microplastics. The numbers preceding PET and PBAT MPs denote the added particle concentrations.

Long-term exposure to PET and PBAT MPs significantly shaped microbial co-occurrence networks and systematically remodeled network topology (Fig. 4a and Supplementary Fig. S8). Compared with the control condition, exposure to PET and PBAT MPs significantly reduced the number of edges and the average degree in the networks. Meanwhile, the decreased clustering coefficients and the increased average path lengths indicated reduced network density, potentially diminishing the efficiency of metabolic cooperation among sewer microorganisms. Modularity increased from 20.6% to 24.5% after PET and PBAT MP exposure, suggesting a shift toward more functionally specialized modules that may exacerbate the vulnerability of microbial communities in sewers.

The null model clarified the role of MPs in shaping community assembly. In the controls, β-nearest taxon index (β-NTI) values clustered predominantly between −2 and +2 (Supplementary Fig. S9), suggesting that stochastic processes (e.g., ecological drift and homogeneous dispersal) primarily governed community dynamics (Fig. 4b). The contribution of deterministic processes (|β-NTI| ≥ 2) increased from 22.2% to 55.5% after long-term exposure to PET MPs, with heterogeneous selection becoming the dominant driver. The PBAT MPs strengthened dispersal limitation, reduced homogeneous dispersal, and increased heterogeneous selection. Together, these shifts indicate that PET and PBAT MPs likely reconfigure ecological niche allocation in sewer microbial communities through selective pressures (e.g., the inhibition of toxicity, resource competition, and the remodeling of co-occurrence networks). In particular, this shift from stochastic to deterministic assembly is consistent with the concept of press disturbance, suggesting that prolonged MP exposure establishes a legacy effect in sewer microbiomes [[47], [48], [49]]. Such deterministic reorganization may introduce hysteresis, preventing communities from fully returning to their original state of stochastic assembly, even after MP stress is alleviated. However, functional redundancy may allow for the partial recovery of metabolic functions.

The deterministic selection pressures and oxidative stress associated with PET and PBAT MPs may have contributed to shifts in the functional structure of the microbial communities forming in the sewer system. Long-term exposure to the PET and PBAT MPs, especially at a concentration of 500 particles L^−1^, significantly altered the relative abundance of key functional microorganisms in the reactors. Sensitive hydrolytic/fermentative bacteria, including *Trichococcus*, *Romboutsia*, and the *Christensenellaceae* R-7 group, decreased by up to 63.4% (Fig. 4c), while tolerant taxa increased. Hydrogen-producing acetogens (HPAs; e.g., an unclassified lineage within the family Synergistaceae, an unclassified lineage affiliated with Bacteroidetes vadinHA17, an unclassified lineage within the order Aminicenantales, and *Smithella*) increased in abundance by up to 63.2% (Fig. 4d). These genera, which catabolize volatile fatty acids and tolerate antibiotics [50,51], likely withstood PET and PBAT MP stress, occupied favorable niches, and performed key metabolic functions in the reactors. The abundance of methanogenic archaea, including *Methanosaeta*, *Methanobacterium*, and *Methanospirillum*, rose significantly by 48.4% to 67.0% (Fig. 4e), which may relate to their metabolic adaptability (e.g., increased abundance of antioxidant genes) [52] and strong resistance to the chemical and physical damage of archaeal cell membranes composed of ether-bonded isoprenoid lipids [53]. Exposure to high MP concentrations decreased the relative abundance of sulfate-reducing bacteria (SRB) by 31.8–49.7%. The metabolically versatile genus *Desulfobulbus*, capable of hydrogenotrophic metabolism via direct interspecies electron transfer or acetate utilization, showed a dose-dependent response. This genus proliferated at low doses but was suppressed at 500 particles L^−1^, with its relative abundance reduced by 38.4–44.3% (Fig. 4f). Collectively, the modularization of cooperative networks, the shift from stochastic to deterministic assembly, and the enrichment of stress-tolerant functional taxa demonstrated that PET and PBAT MPs may have reconfigured the sewer microbial communities. These community-level changes may have driven the reprogramming of core metabolic pathways, which are explored in the subsequent section.

### 3.3 Potential shifts in the key metabolic functions of sewer microbes

#### 3.3.1 Potential disruption of microbial stress response systems and redox signaling pathways

As described above, PET and PBAT MPs promoted the accumulation of intracellular ROS in sewage microorganisms. In addition, the glycine radicals produced during pyruvate oxidation by formate C-acetyltransferase (*pfl*B) may have contributed to endogenous ROS formation by facilitating electron transfer, thereby promoting the production of H_2_O_2_ and ·OH [54,55]. Accordingly, the decrease in the abundance of the *pflB* gene may be associated with increased leakage of glycine radicals and the formation of ROS. The respiratory chain associated with oxidative phosphorylation was another primary endogenous source of ROS. After exposure to PET and PBAT MPs at 500 particles L^−1^, the abundance of genes encoding complex II (*sdh*A) increased, whereas those encoding complex III (*cyd*A and *cyd*B) declined, implying that electron accumulation occurs at ubiquinone carriers in sewer microbes. This redox imbalance may increase the likelihood of electron leakage to O_2_, thereby favoring ROS formation (e.g., O_2_^·−^) [52,56].

**Fig. 5:**
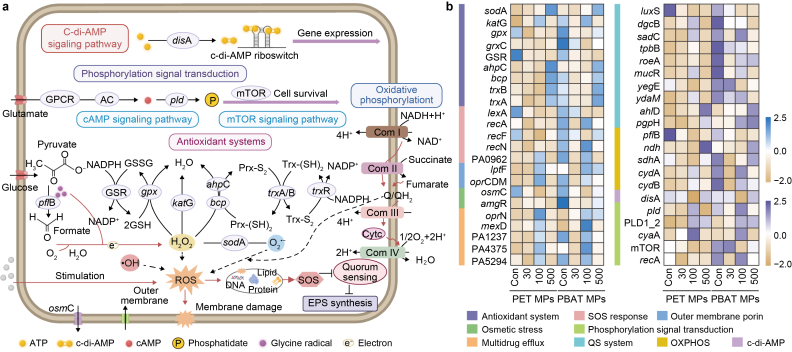
Proposed mechanism of MP-induced oxidative stress and associated functional responses in sewer microbiomes. **a**, Endogenous reactive oxygen species (ROS) generation and microbial metabolism induced by PET and PBAT MPs in sewers. **b**, Functional genes involved in stress-response systems, cyclic diadenosine monophosphate (c-di-AMP) signaling pathway, and phosphorylation signal transduction. MPs: microplastics; cAMP: cyclic adenosine monophosphate; mTOR: mammalian target of rapamycin; GSH: reduced glutathione; GSSG: oxidized glutathione; Prx-(SH)_2_: reduced peroxiredoxin; Prx-S_2_: oxidized peroxiredoxin; Trx-(SH)_2_: reduced thioredoxin; Trx-S_2_: oxidized thioredoxin; ·OH: hydroxyl radicals; O_2_^·−^: superoxide radical anions; EPS: extracellular polymeric substance; QS: quorum sensing; OXPHOS: oxidative phosphorylation; PET: polyethylene terephthalate; PBAT: polybutylene adipate terephthalate. Con, 30, 100, and 500 denote PET and PBAT MP concentrations of 0, 30, 100, and 500 particles L^−1^, respectively.

Upon MP exposure, the abundance of genes involved in microbial antioxidant defense changed, reflecting a shift in microbial capacity to cope with oxidative stress. SOD first converted O_2_^·−^ into H_2_O_2_ and O_2_, while CAT, peroxidase, glutathione peroxidase, and peroxiredoxin (Prx) catalyzed H_2_O_2_ conversion to H_2_O (Fig. 5a) [57]. Exposure to PET and PBAT MPs at 30–500 particles L^−1^ increased the abundance of genes encoding SOD (*sod*A) and Prx (*ahp*C and *bcp*), pointing to elevated microbial functional potential for SOD and thiol-dependent peroxide detoxification (Fig. 5b). Notably, the abundance of the gene encoding CAT (*kat*G) increased at low and medium MP concentrations but declined at higher doses, suggesting that CAT-associated antioxidant potential is impaired under considerable MP exposure. This impairment may be associated with the metabolic shift from CAT-associated detoxification to the higher-affinity Prx/alkyl hydroperoxide reductase system (*ahp*C and *bcp*). In summary, PET and PBAT MP exposure may aggravate oxidative-stress-related functional responses. At high concentrations of such MPs, reduced CAT-associated antioxidant potential may be linked to diminished capacity for redox homeostasis, potentially increasing the likelihood of H_2_O_2_ and organic peroxide accumulation and reducing the ROS-detoxification potential of anaerobic microorganisms.

Beyond aggravating oxidative damage, ROS can act as signaling molecules that regulate cellular responses through multiple pathways, such as cyclic diadenosine monophosphate (c-di-AMP) signaling [58] and phosphorylation signaling [59]. Therefore, PET and PBAT MPs may influence microbial signal transduction. The second messenger c-di-AMP is synthesized from ATP by diadenylate cyclase (*dis*A) [60] and has been associated with osmotic regulation and DNA-damage sensing through interactions with repair-associated receptors [58]. The decrease in *dis*A abundance at low to medium concentrations of PET and PBAT MPs suggests reduced microbial potential for DNA-damage sensing and repair. This pattern possibly reflects a tendency for microorganisms to temporarily restrain cell development and other energy-consuming activities, thereby helping maintain genomic integrity and basic homeostasis under moderate stress [61]. At high PET and PBAT MP concentrations, increased *dis*A abundance suggests enhanced c-di-AMP signaling potential and possible crosstalk among signaling networks, which may affect methanogenic and sulfate-reducing functions [58]. Correspondingly, phosphate-related signal transduction pathways involving the cyclic adenosine monophosphate (cAMP) [62] and mammalian target of rapamycin (mTOR) pathways were evaluated. Extracellular signals can activate cyclases, thereby generating cAMP from ATP, which can subsequently be phosphorylated to phosphatidic acid or processed by phospholipase D (*pld*), thereby activating mTOR signaling [63]. The reduced abundance of these pathway-related genes in sewer microbes exposed to PET and PBAT MPs suggests potential disruptions in carbohydrate metabolism and gene regulation. Because mTOR integrates intracellular and extracellular signals to regulate cellular homeostasis and metabolism [64], the reduced abundance of mTOR-associated genes in this work may reflect decreased signaling potential under exposure to PET and PBAT MPs. Consistent with this phenomenon, changes in the abundance of genes linked to substrate phosphorylation and c-di-AMP signaling point to potential perturbations in microbial signaling and may affect sewer microbial signaling, cell proliferation, and differentiation. While ROS-related effects, together with other stress responses induced by PET and PBAT MPs, may contribute to cellular dysfunction, they may also be associated with signaling processes linked to the persistence or recovery of critical anaerobic microbes such as methanogenic archaea and HPAs. This pattern suggests that these microbes possess adaptive strategies to mitigate metabolic inhibition or to recover metabolic function after stress exposure. Consequently, changes in redox signaling-related functional potential (c-di-AMP and phosphorylation pathways) may help maintain redox homeostasis and improve tolerance to PET and PBAT MP stress. This process may be crucial for detoxification against MP stress in anaerobic microbes.

Meanwhile, the coordinated reduction in critical SOS-response genes (*lex*A, *rec*A/F/N, and PA0962) suggests that exposure to PET and PBAT MPs at 30–500 particles L^−1^ reduces the functional potential for SOS response, mismatch repair, and homologous recombination, potentially contributing to increased genomic instability. Conversely, the abundance of genes encoding outer-membrane proteins (*lpt*F and *opr*CDM) and osmotic-stress regulators (*osm*C) increased following exposure to substantial PET and PBAT MPs (Fig. 5b). Because outer membrane proteins can function as diffusion channels, their increased abundance may be associated with reinforced membrane permeability [65], potentially facilitating the entry of aged constituents of PET and PBAT MPs (e.g., ROS and leachates) into microbial cells. The increased abundance of efflux pump genes (*opr*N, *mex*D, PA1237, PA4375, and PA5294) under low-to-moderate PET and PBAT MP exposure suggests enhanced microbial defense capacity to extrude toxic compounds and alleviate MP stress. However, the abundance of efflux pump genes declined at high doses, potentially reducing the community's potential to eliminate harmful substances and increasing the likelihood of intracellular toxin accumulation and cellular stress. Quorum sensing (QS) coordinates bacterial behavior through signaling molecules such as acyl-homoserine lactones (AHLs) and cyclic di-GMP (c-di-GMP), which regulate metabolic processes, including EPS synthesis [66]. Substantial concentrations of PET and PBAT MPs reduced the abundance of AHL-related genes (*lux*S) and c-di-GMP biosynthesis genes (*dgc*B, *sad*C, *tpb*B, *muc*R, and *yda*M) while increasing the AHL-related and c-di-GMP quorum-quenching genes (*ahl*D and *pgp*H) (Fig. 5b). These findings suggest that exposure to PET and PBAT MPs at 500 particles L^−1^ is associated with reduced QS-related functional potential. Therefore, the increased abundance of stress-related genes at high concentrations of PET and PBAT MPs may represent a compensatory stress response accompanied by broader physiological perturbations [67], including altered redox balance, impaired DNA repair potential, altered membrane properties, intracellular toxin accumulation, and weakened community signaling.

#### 3.3.2 Potential shifts in microbial methane and sulfur metabolism

**Fig. 6:**
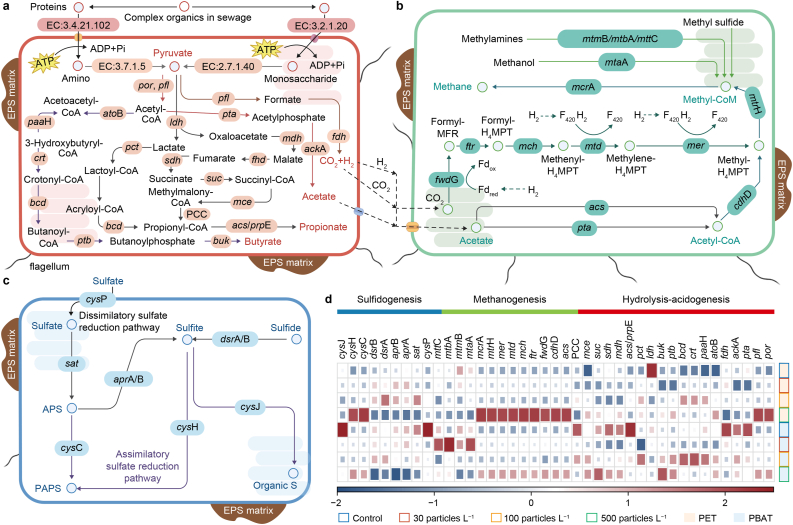
Shifts in carbon, methane, and sulfur metabolism in sewer microbiomes exposed to PET and PBAT MPs. **a**–**c**, Changes in metabolic pathways involved in hydrolysis-acidogenesis (**a**), methanogenesis (**b**), and sulfidogenesis (**c**). **d**, Abundance of genes involved in hydrolysis-acidogenesis, methanogenesis, and sulfidogenesis. PAPS: 3′-phosphoadenosine 5′-phosphosulfate; EPS: extracellular polymeric substance; PET: polyethylene terephthalate; PBAT: polybutylene adipate terephthalate; MPs: microplastics.

Following the identification of PET and PBAT MP-associated imbalances in microbial redox homeostasis, potential implications for the functional potential of sewer microbial communities were investigated. During hydrolytic acidification, sustained exposure to PET and PBAT MPs may have reduced the functional potential for amino acid production (EC:3.4.21.102) and shifted substrate utilization potential from complex organic matter to monosaccharides, possibly favoring pyruvate-producing pathways (EC:3.2.1.20; EC:2.7.1.40) (Supplementary Fig. S10). Additionally, exposure to PET and PBAT MPs at 30–500 particles L^−1^ likely enhanced the potential for formate and butyrate biosynthesis while suppressing acetate production (Fig. 6a–d). This transition hints at microbial redox balancing through a redistribution of metabolic potential [68]. When the conversion of pyruvate to acetate was constrained, increased formate-to-hydrogen conversion may have compensated, stabilizing the NAD^+^/NADH ratio and illustrating metabolic flexibility and redundancy under MP stress. During methanogenesis, the decrease in *pta* gene abundance suggested that exposure to PET and PBAT MPs at 30–500 particles L^−1^ diminished the potential for acetyl-CoA synthesis in methanogenic archaea (Fig. 6b–d). The increased abundance of *cdh*D and *acs* may reflect a compensatory shift response to the reduced potential for acetyl-CoA synthesis. Concurrently, exposure to PET and PBAT MPs at 30–500 particles L^−1^, particularly at 500 particles L^−1^, significantly augmented the abundance of genes associated with hydrogenotrophic methanogenesis (e.g., *fdw*G, *ftr*, *mch*, *mtd*, and *mer*), indicating enhanced microbial potential for H_2_-dependent CO_2_ reduction. These results imply that exposure to PET and PBAT MPs at 30–500 particles L^−1^, especially at 500 particles L^−1^, enhances acetoclastic and hydrogenotrophic methanogenic activity, possibly increasing terminal methane production potential, as reflected by the increased *mcr*A gene abundance observed in this work.

**Fig. 7:**
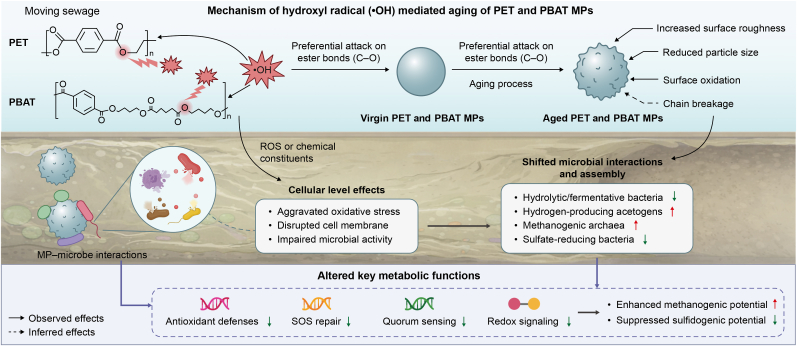
Proposed mechanisms underlying the aging of PET and PBAT MPs and their ecological impacts in sewer systems. Schematic summary of the aging of PET and PBAT MPs in sewers, the associated generation of ROS, and the resulting effects on pollutant transformation, microbial physiology, community assembly, and key metabolic processes in sewer systems. ROS: reactive oxygen species; PET: polyethylene terephthalate; PBAT: polybutylene adipate terephthalate; MPs: microplastics.

Additionally, the abundance of *apr*A/B and *dsr*A/B increased slightly under PET MPs at 30–100 particles L^−1^ but decreased significantly by 40.4–55.5% at 500 particles L^−1^. This suggests reduced functional potential for dissimilatory sulfate reduction under considerable PET MP exposure (Fig. 6c and d). Because *apr*A/B and *dsr*A/B encode the rate-limiting enzymes adenosine-5′-phosphosulfate reductase and dissimilatory sulfate reductase, their decreased abundance implies reduced sulfidogenic potential in the residual SRB community. In contrast, the PBAT MPs may have curtailed sulfide production across all concentrations (30–500 particles L^−1^), possibly due to their inherent chemical properties or the release of constituents during aging. Collectively, these gene-level responses may help explain the reduced sulfide production described in Section 3.2.1. Although high concentrations of PET and PBAT MPs enhanced the abundance of sulfur-assimilation genes (*cys*C and *cys*H), the depletion of gene *cys*J may indicate reduced potential for the conversion of inorganic to organic sulfur. Consequently, substantial exposure to PET and PBAT MPs may suppress the potential for both assimilatory and dissimilatory sulfate reduction, likely lowering the risk of sulfide accumulation in sewer environments. PET and PBAT MPs may shift microbial metabolism toward enhanced methanogenic potential and reduced sulfidogenic potential. Although reduced sulfide production potential may lower odor and corrosion risks, enhanced methanogenic potential may elevate methane emissions and the risk of combustible gas accumulation in poorly ventilated or enclosed sewer segments. These possibilities suggest that MP accumulation represents an emerging stressor that could complicate the simultaneous control of H_2_S and CH_4_, rather than an effective method for sulfide suppression. Collectively, PET and PBAT MPs, associated secondary stressors (e.g., ROS and leachates from aged MPs), and MP–microbe interactions may weaken the functional robustness of sewer microbiomes by contributing to oxidative stress-related disturbances as well as alterations in redox homeostasis and cellular regulation (Fig. 7). Once transported to downstream WWTPs, these stressors may further influence key functional taxa and potentially compromise the stability of biological treatment.

## 4 Conclusion

This study explored the ·OH-driven aging mechanisms of PET and PBAT MPs and their impacts on the microbial ecology and functional potential in sewer systems. ·OH preferentially attacked ester (C–O) bonds in PET and PBAT MPs, ultimately causing chain scission and the formation of hydroxyl and carboxyl oligomers. The MPs, the chemical constituents that they released, and their interactions with microbes may have initially weakened microbial interactions, restructured the microbial communities in our sewer system, and shifted community assembly from stochastic to deterministic processes. In addition, PET and PBAT MPs, particularly at high concentrations, may have altered the functional potential associated with microbial stress-response and signaling processes, including antioxidant defense, SOS response, QS, and redox signaling, possibly enhancing methanogenic potential and eroding sulfidogenic potential. Although these alterations may mitigate the risk of sulfide-induced sewer corrosion, they may also increase methane production potential, which could exacerbate greenhouse gas emissions and raise sewer safety concerns. Moreover, progressive in-sewer aging during transport can increase the specific surface area of MPs and environmental reactivity, potentially amplifying ecotoxicological risks in downstream WWTPs. Therefore, MP management should extend beyond WWTPs to include upstream sewer systems. Efforts should focus on reducing MP inputs at the source (e.g., limiting microfiber release during laundry and pretreatment of industrial effluents rich in MPs) and enhancing capture at key sewer nodes to restrict the downstream transport of “pre-aged” MPs to WWTPs. These measures are essential for effective pollution mitigation and the sustainable operation and maintenance of sewer systems.

## CRediT authorship contribution statement

**Yaxin Wang:** Writing – review & editing, Writing – original draft, Visualization, Methodology, Data curation, Formal analysis, Investigation. **Xiuhong Liu:** Writing – review & editing, Supervision, Resources, Project administration. **Zhipeng Zhang:** Investigation, Conceptualization, Data curation. **Ruxian Jing:** Visualization, Formal analysis, Methodology. **Xiaoyin Zhao:** Conceptualization, Methodology. **Weipeng Han:** Investigation, Conceptualization. **Chenduo Huang:** Visualization, Investigation. **Qing Yang:** Project administration, Funding acquisition, Supervision.

## Declaration of competing interest

The authors declare that they have no known competing financial interests or personal relationships that could have appeared to influence the work reported in this paper.
