# Supplementary material for: In-sewer microplastics drive microbial metabolic shifts toward enhanced methanogenesis
Source: Environ Sci Ecotechnol. 2026 Jun 20;32:100726. doi: 10.1016/j.ese.2026.100726 (PMC13319378; doi:10.1016/j.ese.2026.100726)
Supplement: Multimedia component 1 [file mmc1.docx]

**Supplementary Material for**

**In-sewer microplastics drive microbial metabolic shifts toward enhanced methanogenesis**

Yaxin Wang^1, 2^, Xiuhong Liu^1, *^, Zhipeng Zhang^2^, Ruxian Jing^2^, Xiaoyin Zhao^3^, Weipeng Han^2^, Chenduo Huang^1^, Qing Yang^2, *^

^1^Key Laboratory of Beijing for Water Quality Science and Water Environment Recovery Engineering, Beijing University of Technology, Beijing, 100124, China.

^2^National Engineering Laboratory for Advanced Municipal Wastewater Treatment and Reuse Technology, Beijing University of Technology, Beijing, 100124, China.

^3^Beijing Waterworks Group Co., Ltd, Beijing, 100031, China.

^*^Corresponding author: Xiuhong Liu, Qing Yang

E-mail addresses: lxhfei@163.com, yangqing@bjut.edu.cn

Tel/fax: 86-10-67392070

Postal address: No. 100 Ping Leyuan, Chaoyang District, Beijing, 100124, China

Summary Information

Number of pages: 26

Figures: S1-S10

Tables: S1-S6

Texts: S1-S5

**Texts**

**Text S1. EPS extraction**

Sludge samples were directly collected from the sewer reactor, stored at 4 °C for no longer than 24 h. A portion of each sample was used for the determination of mixed liquor suspended solids (MLSS) and mixed liquor volatile suspended solids (MLVSS) using an ash analyzer (prepASH 212, Precisa Gravimetrics AG, Switzerland). The remaining portion was subjected to ultrasonic disruption at 20 kHz and 50 W. Sonication was performed for 120 s in pulse mode (10 s on, 10 s off) for 3 cycles. After homogenization, the samples were centrifuged at 5000 rpm for 15 min at 4 °C, and the supernatant was subsequently collected and passed through 0.22 μm membranes to obtain EPS.

**Text S2. Analytical methods for ROS, LDH, and the activities of CAT and SOD**

ROS levels and LDH release were detected using ROS assay kit (Shanghai Bioleaper Biotechnology Co., Ltd., China) and enzyme-linked immunosorbent assay (ELISA) kits (Jiangsu Jingmei Biotechnology Co., Ltd., China) following the manufacturer’s instructions, respectively. The activities of CAT and SOD were also determined according to the manufacturer’s protocols (Beijing Solarbio Technology Co., Ltd., China). Briefly, sludge samples were collected directly from the sewer reactor and lyophilized in a freeze-dryer (FreeZone, Labconco, USA) for 72 hours. Weigh approximately 0.5g of the lyophilized sample and add a phosphate-buffered saline (PBS) solution (0.01 mol L^-1^, pH=7.2-7.4) at a weight-to-volume ratio of 1:5. Vortex mix thoroughly for 30 sec. Centrifuge at 4000 rpm for 15 min, and then collect the supernatant for ROS testing. Next, weigh 0.1 g of the sample, add 1 mL of the extraction solution, and homogenize it on ice. Centrifuge at 12000 rpm at 4°C for 10 min, and then collect the supernatant for LDH testing.

**Text S3. Flow cytometry analysis**

The sample pretreatment methods were based on previous research [1]. Briefly, take 500 μL of sludge from the sewer reactor and wash three times with PBS solution (pH=6.8-7.2, 0.1 mol L^-1^). Dilute the mixture tenfold and sonicate for 18 seconds on ice using a 50W ultrasonicator. Subsequently, dilute the disrupted sludge tenfold and filter through a 10 μm membrane. The fluorochromes Propidium Iodide (PI) and SYBR Green I (Shanghai Yuanye Biotechnology Co., Ltd., China) have been widely reported as high-affinity nucleic acid dyes that provide bright cell staining. A 1:100 dilution of the SYBR Green I commercial stock solution was made in DMSO. The PI commercial stock solution has a concentration of 1 mg mL^-1^. The double staining equilibrium was reached within 15 min of incubation at room temperature in the dark. cFDA (Shanghai Aladdin Biochemical Technology Co., Ltd., China) was used to evaluate enzymatic activity. For adequate signals, use 10 μL of a 0.1 mmol L^-1^ cFDA solution per mL of sample. Incubate the samples for 30 minutes at 37°C in the dark. Flow cytometric measurements were performed on a BD FACSAria III flow cytometer. Approximately 100000 events were collected per sample, and the data were analyzed in FlowJo (v10.8.1).

**Text S4. Free radical measurements and EPR parameters**

10 μL of Milli-Q water and sewage of PET and PBAT MPs were placed in centrifuge tubes. Then, either 100 μL DMPO solution (1 mol L^-1^) was added for the detection of ·OH, or 100 μL dimethyl sulfoxide (DMSO) and 100 μL DMPO solution (1 mol L^-1^) were added for the detection of O_2_^·-^. The solution was quickly transferred to a capillary tube, sealed tightly with vacuum grease, inserted securely into a quartz tube, and fastened within the EPR instrument chamber for EPR analysis.

EPR instrument parameters were adjusted as: center field, 3510 G; microwave power, 19.71 mW; sweep width, 100 G; time constant, 10.24 ms; sweep time, 46.08 s.

**Text S5. Quantifying microbial community assembly processes**

To infer the ecological processes shaping the composition of sewer microbial communities, a null-model analysis based on phylogenetic information was applied. The mean nearest taxon distance (MNTD) and its community-level analogue βMNTD were calculated using the R package picante v1.8. By randomizing taxonomic units on the phylogenetic tree (999 randomizations), the standard deviation between observed MNTD/βMNTD and the null model mean was calculated. The picante package further computed the nearest taxon index (NTI) and βNTI to quantify the degree to which local community structure and dynamics deviate from purely random ecological processes [3]. The proportion of pairwise comparisons with |βNTI|>2 indicated selection effects, whereas |βNTI|<2 suggested dominance by random processes [4].

Subsequently, random processes from diffusion-limited were distinguished from homogenizing diffusion and ecological drift by calculating the Raup-Crick metric based on Bray-Curtis distances (RC_bray_). This metric compares observed Bray-Curtis distances with expected distances from 9999 randomized simulations [3]. The relative contributions of ecological processes in each category using distinct criteria were quantified: |βNTI|>2 indicated selection; |βNTI|<2 and RC_bray_>0.95 denoted dispersal limitation; |βNTI|<2 and RC_bray_<-0.95 indicated homogenizing dispersal; |βNTI|<2 and |RC_bray_|<0.95 denoted ecological drift. The selection processes were further divided into homogeneous selection (βNTI<-2) and heterogeneous selection (βNTI>2).

**
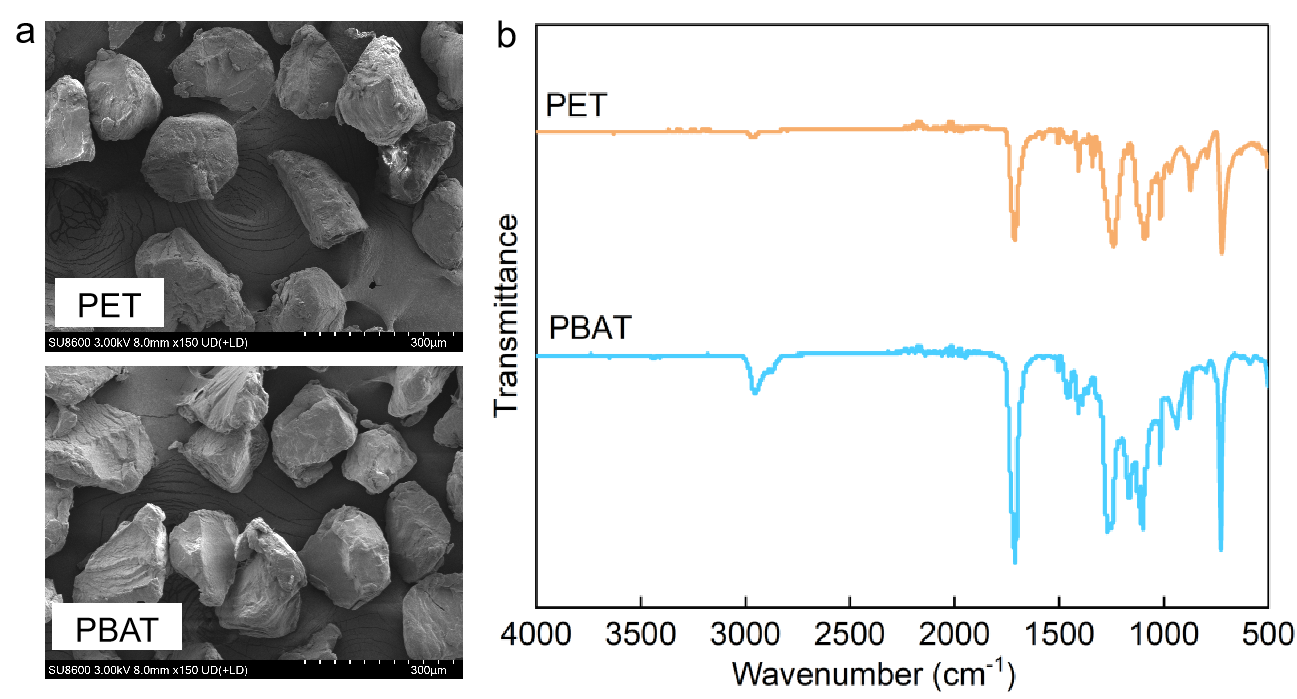
**

**Fig. S1.** (a) SEM micrographs and (b) ATR-FTIR spectra of PET and PBAT MPs.


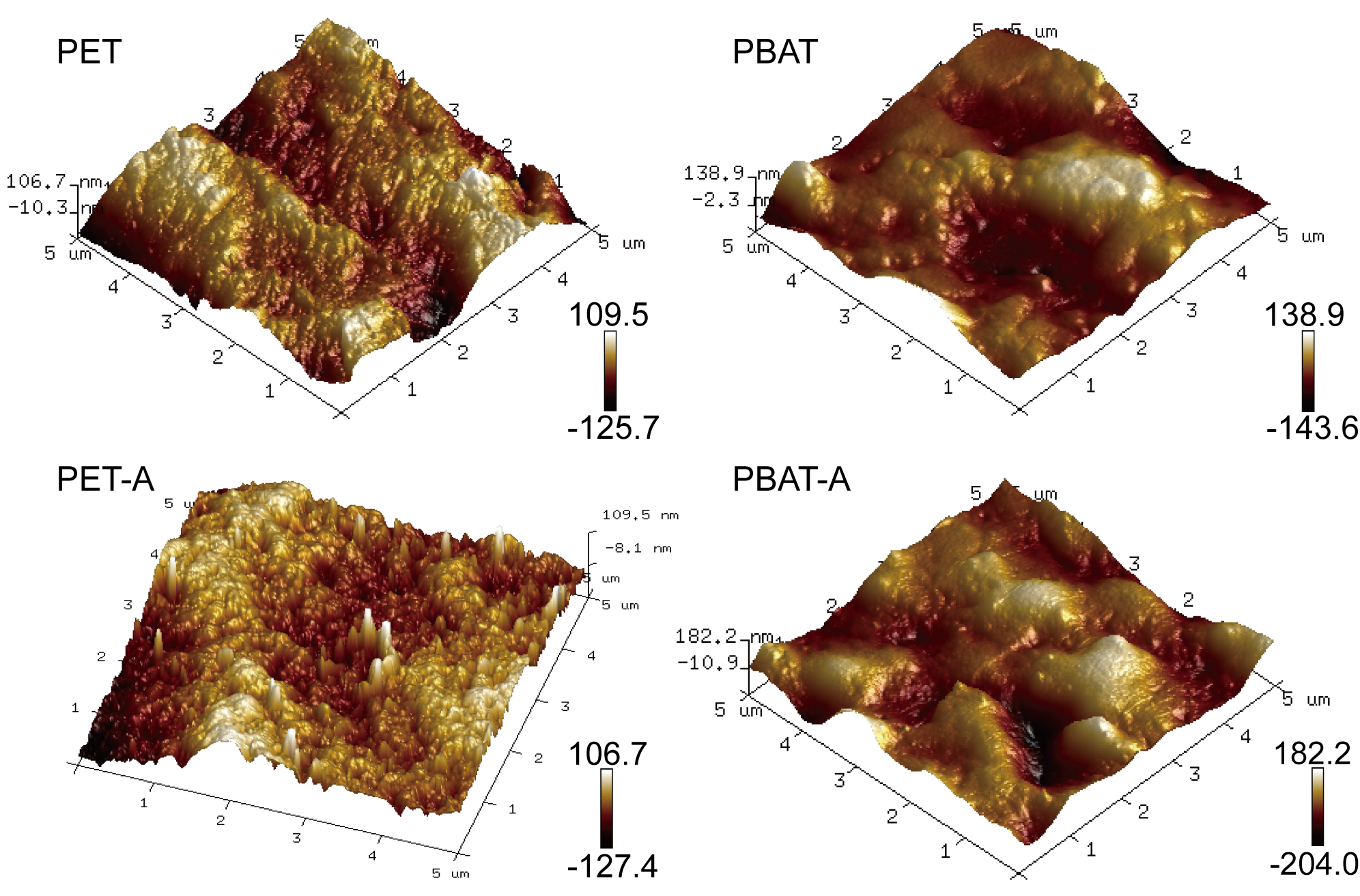


**Fig. S2.** Surface morphology of pristine and aged PET and PBAT MPs. PET-A and PBAT-A represent aged PET MPs and aged PBAT MPs, respectively.


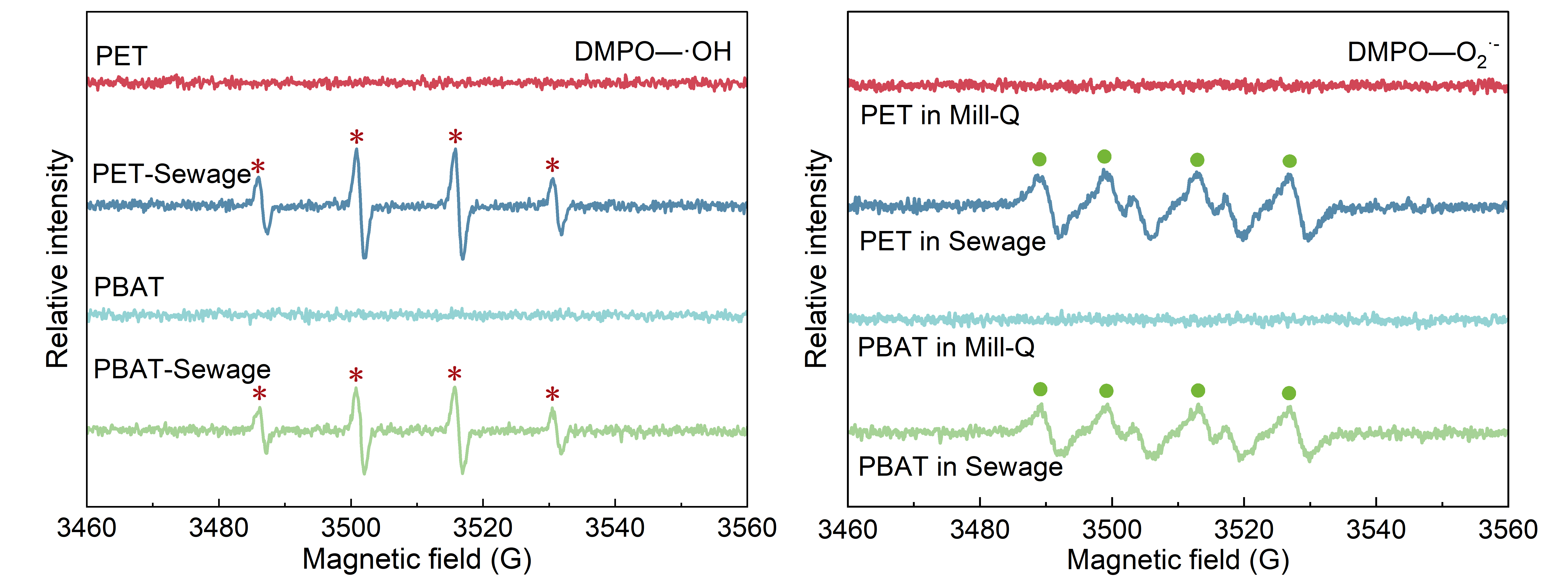


**Fig. S3.** EPR spectra of O_2_^•-^ of PET and PBAT MPs in Milli-Q and sewer sewage.


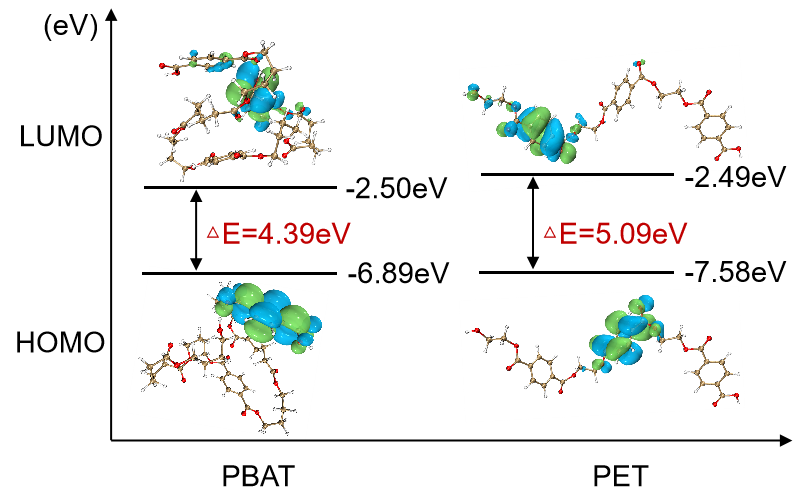


**Fig. S4.** Visualization of LUMO/HOMO plots and molecular orbital energies for PET and PBAT MPs. The green color indicated the positive phase of the wave function, while the blue denoted the negative phase.


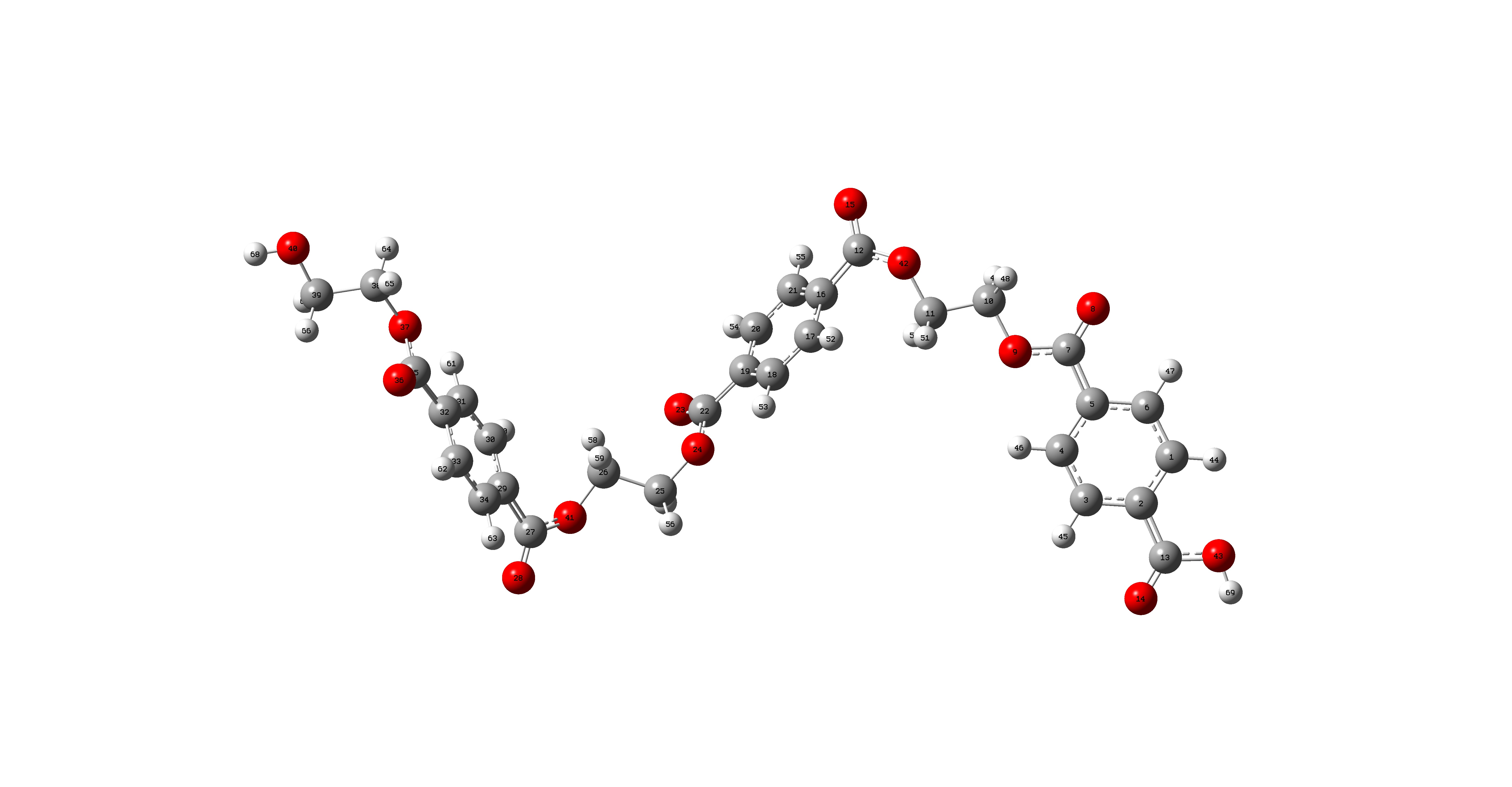


a

b


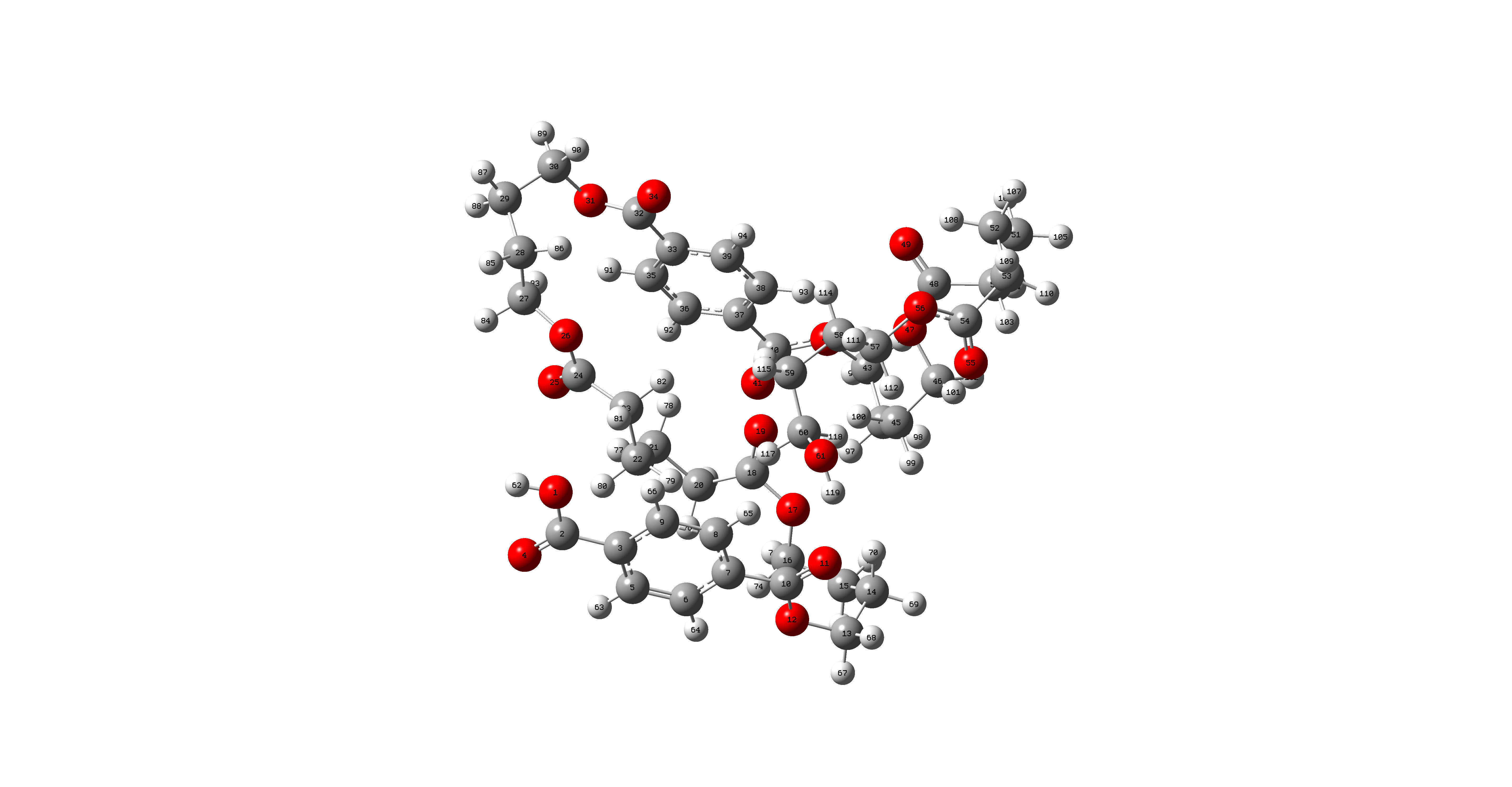


**Fig. S5.** (a) PET and (b) PBAT molecules.


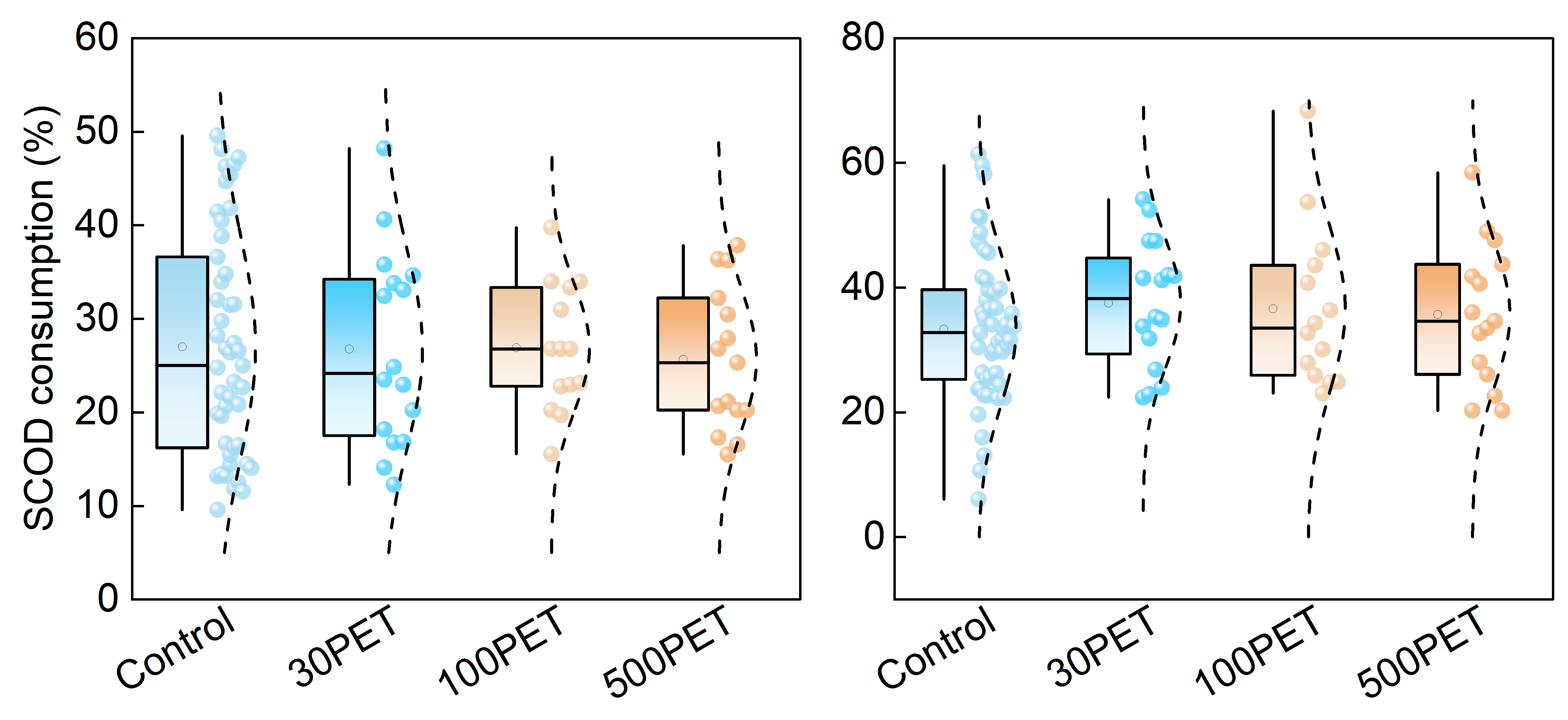


**Fig. S6.** Changes of SCOD exposed to PET and PBAT MPs.





**Fig. S7.** Changes of NH_4_^+^-N and PO_4_^3-^-P exposed to (a) PET and (b) PBAT MPs.

**Fig. S8.** Co-occurrence networks of genera of microbial community in sewers.


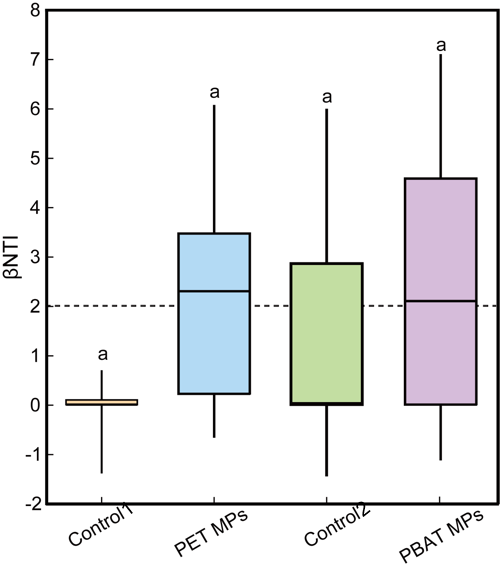


**Fig. S9.** Comparison of β-NTI across different groups. Control1: the control corresponding to the PET MP exposure; Control2: the control corresponding to the PBAT MP exposure.


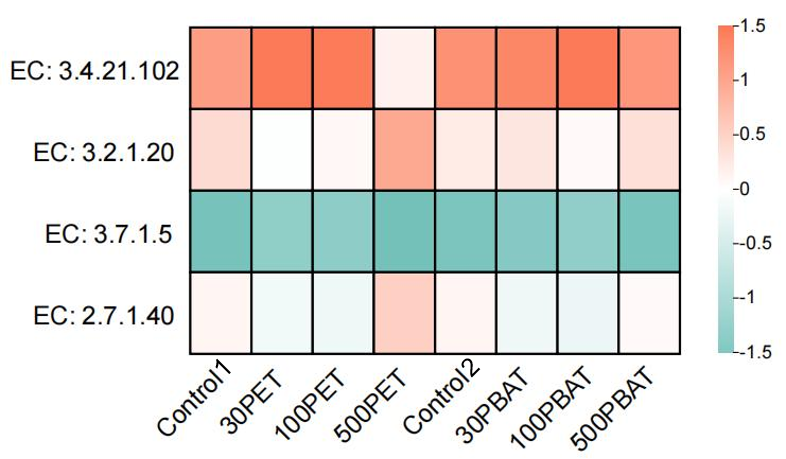


**Fig. S10.** Abundance of enzymes involved in the degradation of complex organics. Control1: the control corresponding to the PET MP exposure; Control2: the control corresponding to the PBAT MP exposure.**Table S1**. Quality of actual domestic sewage.

| Parameters | SCOD (mg L^-1^) | PO_4_^3-^-P (mg L^-1^) | NH₄^+^-N (mg L^-1^) | SO_4_²^-^ (mg L^-1^) |
| --- | --- | --- | --- | --- |
|  |  |  |  |  |
| Contents | ~350 | ~7 | ~60 | ~50 |

#

**Table S2.** Network analysis based on the Spearman correlation coefficients was visualized by Gephi (*p*<0.05).

| Substrate type | PET-Control  (top 50 genera, \|r\|>0.7) | PET  (top 50 genera, \|r\|>0.7) | PET-Control  (top 50 genera, \|r\|>0.9) | PET  (top 50 genera, \|r\|>0.9) | PET-Control  (top 100 genera, \|r\|>0.8) | PET  (top 100 genera, \|r\|>0.8) |
| --- | --- | --- | --- | --- | --- | --- |
| Number of nodes | 50 | 49 | 50 | 32 | 99 | 98 |
| Number of edges | 502 | 99 | 502 | 40 | 1901 | 512 |
| Positive correlation | 63.35% | 54.55% | 63.35% | 67.5% | 68.33% | 55.27% |
| Negative correlation | 36.65% | 45.45% | 36.65% | 32.5% | 31.67% | 44.73% |
| Average degree | 20.08 | 4.041 | 20.08 | 2.5 | 38.404 | 10.449 |
| Average clustering coefficient | 1 | 0.538 | 1 | 0.617 | 1 | 0.514 |
| Average path length | 1 | 3.867 | 1 | 1.877 | 1 | 2.85 |
| Modularity | 0.437 | 0.57 | 0.437 | 0.658 | 0.344 | 0.424 |
| Substrate type | PBAT-Control  (top 50 genera, \|r\|>0.7) | PBAT  (top 50 genera, \|r\|>0.7) | PBAT-Control  (top 50 genera, \|r\|>0.9) | PBAT  (top 50 genera, \|r\|>0.9) | PBAT-Control  (top 100 genera, \|r\|>0.8) | PBAT  (top 100 genera, \|r\|>0.8) |
| Number of nodes | 49 | 49 | 49 | 49 | 99 | 99 |
| Number of edges | 510 | 105 | 510 | 105 | 1873 | 456 |
| Positive correlation | 60.78% | 51.43% | 60.78% | 51.43% | 56.06% | 50% |
| Negative correlation | 39.22% | 48.57% | 39.22% | 48.57% | 43.94% | 50% |
| Average degree | 20.816 | 4.286 | 20.816 | 4.286 | 37.838 | 9.212 |
| Average clustering coefficient | 1 | 1 | 1 | 1 | 1 | 1 |
| Average path length | 1 | 1 | 1 | 1 | 1 | 1 |
| Modularity | 0.502 | 0.845 | 0.502 | 0.845 | 0.496 | 0.816 |

# **Table S3.** The sign of each cross-peaks in synchronous and asynchronous maps of PET MPs based on 2D-COS analysis.

| **Peak (cm^–1^)** | **Band assignments** | **Sign** | | | | | |
| --- | --- | --- | --- | --- | --- | --- | --- |
|  |  | 720 | 1020 | 1100 | 1240 | 1410 | 1710 |
| 720 | CH_2_ in plane wobble vibration | + | +(-) | +(-) | +(+) | +(-) | +(+) |
| 1020 | C-O |  | + | +(-) | +(+) | +(-) | +(+) |
| 1100 | C-H |  |  | + |  | +(+) | +(+) |
| 1240 | C-O |  |  |  | + | +(-) | +(-) |
| 1410 | CH_2_ |  |  |  |  | + | +(+) |
| 1710 | C=O |  |  |  |  |  | + |

# **Table S4.** The sign of each cross-peaks in synchronous and asynchronous maps of PBAT MPs based on 2D-COS analysis.

| **Peak (cm^–1^)** | **Band assignments** | **Sign** | | | | |
| --- | --- | --- | --- | --- | --- | --- |
|  |  | 720 | 1020 | 1100 | 1260 | 1710 |
| 720 | CH_2_ in plane wobble vibration | + | +(+) | +(+) | +(+) | +(-) |
| 1020 | C-O |  | + | +(-) | +(-) | +(-) |
| 1100 | C-O-C |  |  | + | +(-) | +(-) |
| 1260 | C-O-C |  |  |  | + | +(-) |
| 1710 | C=O |  |  |  |  | + |

**Table S5.** Partial calculation results of the Fukui function for PET MPs.

| **Atom** | **q(N)** | **q(N-1)** | **q(N+1)** | **f-** | **f+** | **f0** |
| --- | --- | --- | --- | --- | --- | --- |
| 28(O ) | -0.2546 | -0.2885 | -0.1898 | 0.0648 | 0.0339 | 0.0493 |
| 15(O ) | -0.2516 | -0.2849 | -0.1988 | 0.0528 | 0.0333 | 0.043 |
| 6(C ) | -0.0219 | -0.0434 | 0.0209 | 0.0429 | 0.0214 | 0.0322 |
| 3(C ) | -0.0223 | -0.0417 | 0.0186 | 0.0409 | 0.0195 | 0.0302 |
| 40(O ) | -0.2387 | -0.2446 | -0.1846 | 0.0541 | 0.0059 | 0.03 |
| 14(O ) | -0.2745 | -0.3096 | -0.2527 | 0.0218 | 0.0352 | 0.0285 |
| 36(O ) | -0.26 | -0.292 | -0.2353 | 0.0247 | 0.032 | 0.0283 |
| 32(C ) | -0.0077 | -0.0333 | 0.0217 | 0.0294 | 0.0256 | 0.0275 |
| 8(O ) | -0.2577 | -0.2916 | -0.2369 | 0.0207 | 0.0339 | 0.0273 |
| 2(C ) | -0.0061 | -0.034 | 0.0154 | 0.0215 | 0.0279 | 0.0247 |
| 23(O ) | -0.2568 | -0.2876 | -0.2422 | 0.0146 | 0.0308 | 0.0227 |
| 29(C ) | -0.0059 | -0.0274 | 0.018 | 0.0239 | 0.0215 | 0.0227 |
| 1(C ) | -0.0255 | -0.0487 | -0.0042 | 0.0213 | 0.0232 | 0.0223 |
| 5(C ) | -0.0071 | -0.0318 | 0.0111 | 0.0182 | 0.0247 | 0.0214 |
| 19(C ) | -0.0088 | -0.0307 | 0.01 | 0.0188 | 0.0219 | 0.0204 |
| 34(C ) | -0.0231 | -0.0415 | -0.0012 | 0.022 | 0.0184 | 0.0202 |
| 16(C ) | -0.004 | -0.0276 | 0.0123 | 0.0163 | 0.0236 | 0.0199 |
| 31(C ) | -0.0279 | -0.0465 | -0.0066 | 0.0212 | 0.0186 | 0.0199 |
| 27(C ) | 0.2094 | 0.1903 | 0.227 | 0.0176 | 0.0191 | 0.0184 |
| 21(C ) | -0.0213 | -0.0401 | -0.0034 | 0.0179 | 0.0188 | 0.0183 |
| 33(C ) | -0.0205 | -0.0406 | -0.0061 | 0.0144 | 0.0201 | 0.0173 |
| 4(C ) | -0.0294 | -0.0473 | -0.0129 | 0.0166 | 0.0178 | 0.0172 |
| 12(C ) | 0.2103 | 0.192 | 0.2247 | 0.0144 | 0.0183 | 0.0163 |
| 13(C ) | 0.2097 | 0.1839 | 0.2163 | 0.0066 | 0.0258 | 0.0162 |
| 18(C ) | -0.0277 | -0.0449 | -0.0128 | 0.0149 | 0.0172 | 0.016 |
| 20(C ) | -0.0191 | -0.0389 | -0.009 | 0.0101 | 0.0198 | 0.015 |
| 47(H ) | 0.0536 | 0.0406 | 0.0695 | 0.016 | 0.0129 | 0.0145 |
| 35(C ) | 0.2092 | 0.1867 | 0.2156 | 0.0064 | 0.0225 | 0.0144 |
| 43(O ) | -0.1727 | -0.1912 | -0.1624 | 0.0102 | 0.0185 | 0.0144 |
| 30(C ) | -0.0308 | -0.0459 | -0.0174 | 0.0133 | 0.0151 | 0.0142 |
| 44(H ) | 0.0505 | 0.0363 | 0.0643 | 0.0138 | 0.0141 | 0.014 |
| 7(C ) | 0.2098 | 0.1869 | 0.2148 | 0.0051 | 0.0229 | 0.014 |
| 22(C ) | 0.2102 | 0.1879 | 0.2139 | 0.0037 | 0.0223 | 0.013 |
| 41(O ) | -0.1305 | -0.1412 | -0.1155 | 0.015 | 0.0107 | 0.0129 |
| 45(H ) | 0.0531 | 0.0418 | 0.0675 | 0.0145 | 0.0113 | 0.0129 |
| 17(C ) | -0.0306 | -0.0467 | -0.0217 | 0.009 | 0.016 | 0.0125 |
| 42(O ) | -0.13 | -0.1412 | -0.1172 | 0.0128 | 0.0113 | 0.012 |
| 62(H ) | 0.0541 | 0.0418 | 0.0656 | 0.0116 | 0.0123 | 0.0119 |
| 63(H ) | 0.0547 | 0.0432 | 0.0668 | 0.0121 | 0.0115 | 0.0118 |
| 68(H ) | 0.1719 | 0.1661 | 0.1897 | 0.0178 | 0.0058 | 0.0118 |
| 69(H ) | 0.1899 | 0.1763 | 0.1997 | 0.0098 | 0.0136 | 0.0117 |
| 61(H ) | 0.049 | 0.0381 | 0.0604 | 0.0113 | 0.011 | 0.0112 |
| 55(H ) | 0.0562 | 0.0439 | 0.066 | 0.0098 | 0.0123 | 0.0111 |
| 54(H ) | 0.055 | 0.0431 | 0.0636 | 0.0086 | 0.0119 | 0.0102 |
| 67(H ) | 0.0258 | 0.0233 | 0.0422 | 0.0164 | 0.0025 | 0.0095 |
| 60(H ) | 0.0498 | 0.0413 | 0.0593 | 0.0095 | 0.0085 | 0.009 |
| 46(H ) | 0.0462 | 0.0377 | 0.0551 | 0.0088 | 0.0085 | 0.0087 |
| 53(H ) | 0.0488 | 0.0391 | 0.0563 | 0.0075 | 0.0097 | 0.0086 |
| 52(H ) | 0.0492 | 0.0393 | 0.0563 | 0.0071 | 0.0099 | 0.0085 |
| 37(O ) | -0.1185 | -0.1281 | -0.1128 | 0.0057 | 0.0096 | 0.0076 |
| 39(C ) | 0.021 | 0.0189 | 0.0335 | 0.0125 | 0.0021 | 0.0073 |
| 24(O ) | -0.1189 | -0.1285 | -0.1146 | 0.0043 | 0.0096 | 0.0069 |
| 48(H ) | 0.0479 | 0.0403 | 0.0538 | 0.0059 | 0.0076 | 0.0068 |
| 66(H ) | 0.0312 | 0.0301 | 0.0435 | 0.0123 | 0.0011 | 0.0067 |
| 64(H ) | 0.0471 | 0.0413 | 0.0546 | 0.0075 | 0.0058 | 0.0066 |
| 57(H ) | 0.0471 | 0.0406 | 0.0534 | 0.0062 | 0.0065 | 0.0064 |
| 56(H ) | 0.0492 | 0.0422 | 0.0541 | 0.0049 | 0.007 | 0.0059 |

**Table S6.** Partial calculation results of the Fukui function for PBAT MPs.

| **Atom** | **q(N)** | **q(N-1)** | **q(N+1)** | **f-** | **f+** | **f0** |
| --- | --- | --- | --- | --- | --- | --- |
| 61(O ) | -0.2359 | -0.243 | -0.1545 | 0.0815 | 0.0071 | 0.0443 |
| 4(O ) | -0.2713 | -0.3313 | -0.2578 | 0.0135 | 0.06 | 0.0368 |
| 33(C ) | -0.0102 | -0.0312 | 0.0287 | 0.0389 | 0.021 | 0.0299 |
| 37(C ) | -0.0017 | -0.0216 | 0.0342 | 0.036 | 0.0198 | 0.0279 |
| 3(C ) | 0 | -0.0471 | 0.0063 | 0.0063 | 0.0471 | 0.0267 |
| 34(O ) | -0.2606 | -0.2878 | -0.2353 | 0.0253 | 0.0271 | 0.0262 |
| 11(O ) | -0.2305 | -0.2864 | -0.2344 | -0.0039 | 0.0559 | 0.026 |
| 10(C ) | 0.2184 | 0.1705 | 0.2197 | 0.0014 | 0.0479 | 0.0246 |
| 41(O ) | -0.2771 | -0.298 | -0.2491 | 0.028 | 0.0209 | 0.0244 |
| 5(C ) | -0.0193 | -0.0529 | -0.0048 | 0.0145 | 0.0336 | 0.024 |
| 7(C ) | -0.0065 | -0.0526 | -0.0051 | 0.0014 | 0.0461 | 0.0238 |
| 6(C ) | -0.0291 | -0.0668 | -0.0201 | 0.009 | 0.0377 | 0.0233 |
| 25(O ) | -0.2642 | -0.2712 | -0.2258 | 0.0384 | 0.007 | 0.0227 |
| 2(C ) | 0.2117 | 0.1697 | 0.2133 | 0.0015 | 0.0421 | 0.0218 |
| 49(O ) | -0.2621 | -0.2622 | -0.2207 | 0.0414 | 0.0001 | 0.0207 |
| 9(C ) | -0.0178 | -0.0533 | -0.0136 | 0.0043 | 0.0355 | 0.0199 |
| 8(C ) | -0.0151 | -0.0504 | -0.0123 | 0.0028 | 0.0353 | 0.0191 |
| 35(C ) | -0.03 | -0.0458 | -0.0079 | 0.0221 | 0.0159 | 0.019 |
| 36(C ) | -0.0228 | -0.0365 | -0.0035 | 0.0193 | 0.0137 | 0.0165 |
| 55(O ) | -0.2644 | -0.2627 | -0.23 | 0.0344 | -0.0017 | 0.0164 |
| 38(C ) | -0.0184 | -0.0331 | -0.0019 | 0.0165 | 0.0147 | 0.0156 |
| 1(O ) | -0.1671 | -0.1948 | -0.1653 | 0.0017 | 0.0278 | 0.0148 |
| 39(C ) | -0.0202 | -0.0362 | -0.0065 | 0.0137 | 0.0159 | 0.0148 |
| 63(H ) | 0.0537 | 0.033 | 0.0621 | 0.0084 | 0.0207 | 0.0145 |
| 64(H ) | 0.0448 | 0.0235 | 0.0516 | 0.0068 | 0.0214 | 0.0141 |
| 32(C ) | 0.2108 | 0.1906 | 0.2169 | 0.0061 | 0.0203 | 0.0132 |
| 62(H ) | 0.1916 | 0.171 | 0.1967 | 0.0051 | 0.0206 | 0.0129 |
| 12(O ) | -0.1087 | -0.1288 | -0.1034 | 0.0053 | 0.0201 | 0.0127 |
| 66(H ) | 0.0534 | 0.0329 | 0.0567 | 0.0033 | 0.0204 | 0.0119 |
| 91(H ) | 0.0458 | 0.0361 | 0.0589 | 0.0132 | 0.0097 | 0.0114 |
| 40(C ) | 0.2106 | 0.194 | 0.2167 | 0.006 | 0.0166 | 0.0113 |
| 92(H ) | 0.0509 | 0.0421 | 0.064 | 0.0131 | 0.0089 | 0.011 |
| 119(H ) | 0.1299 | 0.129 | 0.1494 | 0.0195 | 0.0009 | 0.0102 |
| 118(H ) | 0.0234 | 0.0202 | 0.0402 | 0.0169 | 0.0032 | 0.01 |
| 67(H ) | 0.0416 | 0.0292 | 0.0487 | 0.0071 | 0.0124 | 0.0098 |
| 94(H ) | 0.0522 | 0.0424 | 0.0618 | 0.0096 | 0.0097 | 0.0097 |
| 69(H ) | 0.0406 | 0.0281 | 0.0465 | 0.006 | 0.0124 | 0.0092 |
| 89(H ) | 0.041 | 0.0322 | 0.0502 | 0.0092 | 0.0088 | 0.009 |
| 87(H ) | 0.0382 | 0.0298 | 0.0476 | 0.0094 | 0.0084 | 0.0089 |
| 98(H ) | 0.0286 | 0.0211 | 0.039 | 0.0104 | 0.0074 | 0.0089 |
| 107(H ) | 0.0335 | 0.0286 | 0.0459 | 0.0125 | 0.0049 | 0.0087 |
| 31(O ) | -0.1154 | -0.1246 | -0.1077 | 0.0077 | 0.0092 | 0.0085 |
| 60(C ) | 0.0176 | 0.0186 | 0.0354 | 0.0178 | -0.001 | 0.0084 |
| 56(O ) | -0.1227 | -0.1257 | -0.109 | 0.0136 | 0.003 | 0.0083 |
| 42(O ) | -0.1007 | -0.11 | -0.0937 | 0.0071 | 0.0092 | 0.0082 |
| 117(H ) | 0.0087 | 0.0161 | 0.0321 | 0.0235 | -0.0074 | 0.008 |
| 72(H ) | 0.0353 | 0.028 | 0.0431 | 0.0078 | 0.0073 | 0.0076 |
| 105(H ) | 0.0273 | 0.0226 | 0.0379 | 0.0106 | 0.0047 | 0.0076 |
| 65(H ) | 0.0466 | 0.0306 | 0.0455 | -0.0012 | 0.0161 | 0.0074 |
| 109(H ) | 0.0415 | 0.0379 | 0.0526 | 0.0112 | 0.0035 | 0.0074 |
| 95(H ) | 0.0366 | 0.0309 | 0.0452 | 0.0086 | 0.0057 | 0.0072 |
| 54(C ) | 0.2155 | 0.2134 | 0.2274 | 0.012 | 0.0021 | 0.007 |
| 104(H ) | 0.0413 | 0.0383 | 0.0522 | 0.011 | 0.003 | 0.007 |
| 93(H ) | 0.0463 | 0.0386 | 0.0523 | 0.006 | 0.0077 | 0.0069 |
| 96(H ) | 0.036 | 0.0285 | 0.0423 | 0.0063 | 0.0075 | 0.0069 |
| 77(H ) | 0.0319 | 0.0252 | 0.0387 | 0.0068 | 0.0067 | 0.0068 |

**References:**

[1] G. Ziglio, G. Andreottola, S. Barbesti, G. Boschetti, L. Bruni, P. Foladori, R. Villa, Assessment of activated sludge viability with flow cytometry, Water Res. 36 (2002), 460-468.

[2] X. Zhou, J.T. Lennon, X. Lu, A. Ruan, Anthropogenic activities mediate stratification and stability of microbial communities in freshwater sediments, Microbiome 11 (2023), 191.

[3] J.C. Stegen, X. Lin, J.K. Fredrickson, X. Chen, D.W. Kennedy, C.J. Murray, M.L. Rockhold, A. Konopka, Quantifying community assembly processes and identifying features that impose them, ISME J. 7 (2013), 2069-2079.

[4] J.C. Stegen, X. Lin, A.E. Konopka, J.K. Fredrickson, Stochastic and deterministic assembly processes in subsurface microbial communities, ISME J. 6 (2012), 1653-1664.
